# Uncertainties in projections of sandy beach erosion due to sea level rise: an analysis at the European scale

**DOI:** 10.1038/s41598-020-68576-0

**Published:** 2020-07-17

**Authors:** Panagiotis Athanasiou, Ap van Dongeren, Alessio Giardino, Michalis I. Vousdoukas, Roshanka Ranasinghe, Jaap Kwadijk

**Affiliations:** 1 Deltares, PO Box 177, 2600 MH Delft, The Netherlands; 2 Water Engineering and Management, Faculty of Engineering Technology, University of Twente, PO Box 217, 7500 AE Enschede, the Netherlands; 3 European Commission, Joint Research Centre (JRC), Via Enrico Fermi 2749, 21027 Ispra, Italy; 4 Department of Water Science and Engineering, IHE Delft Institute for Water Education, PO Box 3015, 2610 DA Delft, The Netherlands

**Keywords:** Environmental impact, Environmental sciences, Natural hazards

## Abstract

Sea level rise (SLR) will cause shoreline retreat of sandy coasts in the absence of sand supply mechanisms. These coasts have high touristic and ecological value and provide protection of valuable infrastructures and buildings to storm impacts. So far, large-scale assessments of shoreline retreat use specific datasets or assumptions for the geophysical representation of the coastal system, without any quantification of the effect that these choices might have on the assessment. Here we quantify SLR driven potential shoreline retreat and consequent coastal land loss in Europe during the twenty-first century using different combinations of geophysical datasets for (a) the location and spatial extent of sandy beaches and (b) their nearshore slopes. Using data-based spatially-varying nearshore slope data, a European averaged SLR driven median shoreline retreat of 97 m (54 m) is projected under RCP 8.5 (4.5) by year 2100, relative to the baseline year 2010. This retreat would translate to 2,500 km^2^ (1,400 km^2^) of coastal land loss (in the absence of ambient shoreline changes). A variance-based global sensitivity analysis indicates that the uncertainty associated with the choice of geophysical datasets can contribute up to 45% (26%) of the variance in coastal land loss projections for Europe by 2050 (2100). This contribution can be as high as that associated with future mitigation scenarios and SLR projections.

## Introduction

The global society’s response to impending climate change impacts will be one of the great challenges in the coming decades^1^. With future projections indicating accelerating rates of sea level rise^2,3^, increasing intensity and frequency of extreme sea levels^4–6^ and increases in the exposed population and capital^7^, low elevation coastal zones will be particularly vulnerable to climate change. About 10% of the world’s population resides in these low elevation coastal zones, defined as the area with an elevation less than 10 m above mean sea level^8^.

Coastal zones with sandy beaches, which comprise almost one third of the world’s coastline^9^, are geomorphologically highly dynamic, responding to hydro-morphological processes acting at different time-scales, such as waves, tides, storms and long-term changes of water levels. In particular, sea level rise (SLR) is expected to contribute significantly to future shoreline retreat^10,11^. Shoreline retreat can diminish the touristic and recreational value of beaches and/or cause direct impacts to infrastructure by structural failure. Additionally, since sandy beaches act as the first buffer against storm surges and wave attack, shoreline retreat can increase the vulnerability of the hinterland to flooding^12–15^.

Many studies have taken up the challenge of quantifying shoreline retreat at the local or sub-regional (~ < 500 km of coastline) scale, mostly at locations where high resolution and high accuracy data are available^16–20^. The availability of such data enables the use of higher complexity process models^21,22^, probabilistic assessments^19,23,24^ and uncertainty decomposition^25^. On the other hand, when shoreline retreat is assessed at the regional^26–28^ (~ > 500 km of coastline), continental^29^ or global scale^30,31^, a high number of constraints emerge related to data availability, data resolution, data accuracy, and computational demands^32,33^. This necessitates the use of simpler behavioural models to predict the response of sandy coastlines to SLR at these larger scales. The Bruun Rule^34^ currently offers the only computationally viable method to assess the retreat of sandy shorelines due to SLR at the global, continental or regional scale, even though so far it has received a lot of criticism on its assumptions and widespread use (see Discussion section). This simple two-dimensional mass conservation principle predicts a landward retreat of the shoreline in response to SLR assuming that the beach will maintain an equilibrium profile. It requires as input the SLR amplitude and the nearshore bed slope of the sandy beach. The Bruun Rule has been widely used to derive shoreline retreat projections at the regional^27,28,35^, global^30,31^ and local scale.

Hinkel et al.^30^ assessed the global impacts of SLR using the Bruun Rule in an—at that time—state of the art global framework. They described the spatial distribution of sandy coastlines qualitatively for coastal segments of varying length based on an aggregation of various coastal typology datasets. Additionally, they assumed the same nearshore slope (1:100) across all the sandy beaches globally, neglecting the inherent spatial variability of coastal profile slopes, an assumption that can be quite crucial, since shoreline retreat is linearly dependent on the nearshore slope under the Bruun rule.

In our study, we focus only in SLR driven future shoreline retreat and land loss at sandy beaches, not taking into account other processes driving long-term shoreline change such as natural gradients in alongshore sediment transport, changes in sediment transport due to human interventions (e.g. port construction)^22^, fluvial sediment supply^36,37^, marine feeding^38^ or sand nourishments/land reclamations^9^. These long-term shoreline change patterns are defined herein as ambient shoreline changes^31,39^. Since these patterns might be affected by non-monotonic signals, in our analysis we do not consider these, but rather focus on the effects of SLR alone as an indicator of climate change erosion impact which allows studying uncertainties related to input geophysical datasets. In order to estimate SLR driven shoreline retreat and land loss at sandy beaches (hereafter referred to as coastal land loss for convenience), we also employ the Bruun Rule, due to the scale of the study (i.e. Europe). However, we improve the representation of the geophysical information by computing the shoreline retreat due to SLR at the European scale and its uncertainties using: (a) two different datasets that describe the spatial distribution of sandy beaches, and (b) two different approaches to describe the nearshore slope. For the former, a reclassification from the EUROSION project^40^ and a dataset of satellite derived sandy beaches^9^ (SDSB) are used. For the latter, we apply a newly produced global dataset with spatially varying nearshore slopes^41^ (SVNS) and for comparison purposes, the commonly-used constant slope assumption of 1:100, given that it has been used before in large-scale coastal studies in absence of any other information^29,30^ (see “Methods”, subsection 2). Regional SLR projections associated with the moderate-emission-mitigation-policy scenario (RCP 4.5) and a high-end, business-as-usual scenario (RCP 8.5) assuming also an increased contribution from ice-sheets (as used in Vousdoukas et al.^31^) are applied as boundary conditions^42^. These projections include a glacial isostatic adjustment (GIA) model^43^ and are probabilistic, taking into account uncertainties related to SLR physical processes and climate modelling (see “Methods”, subsection 3).

**Figure 1:**
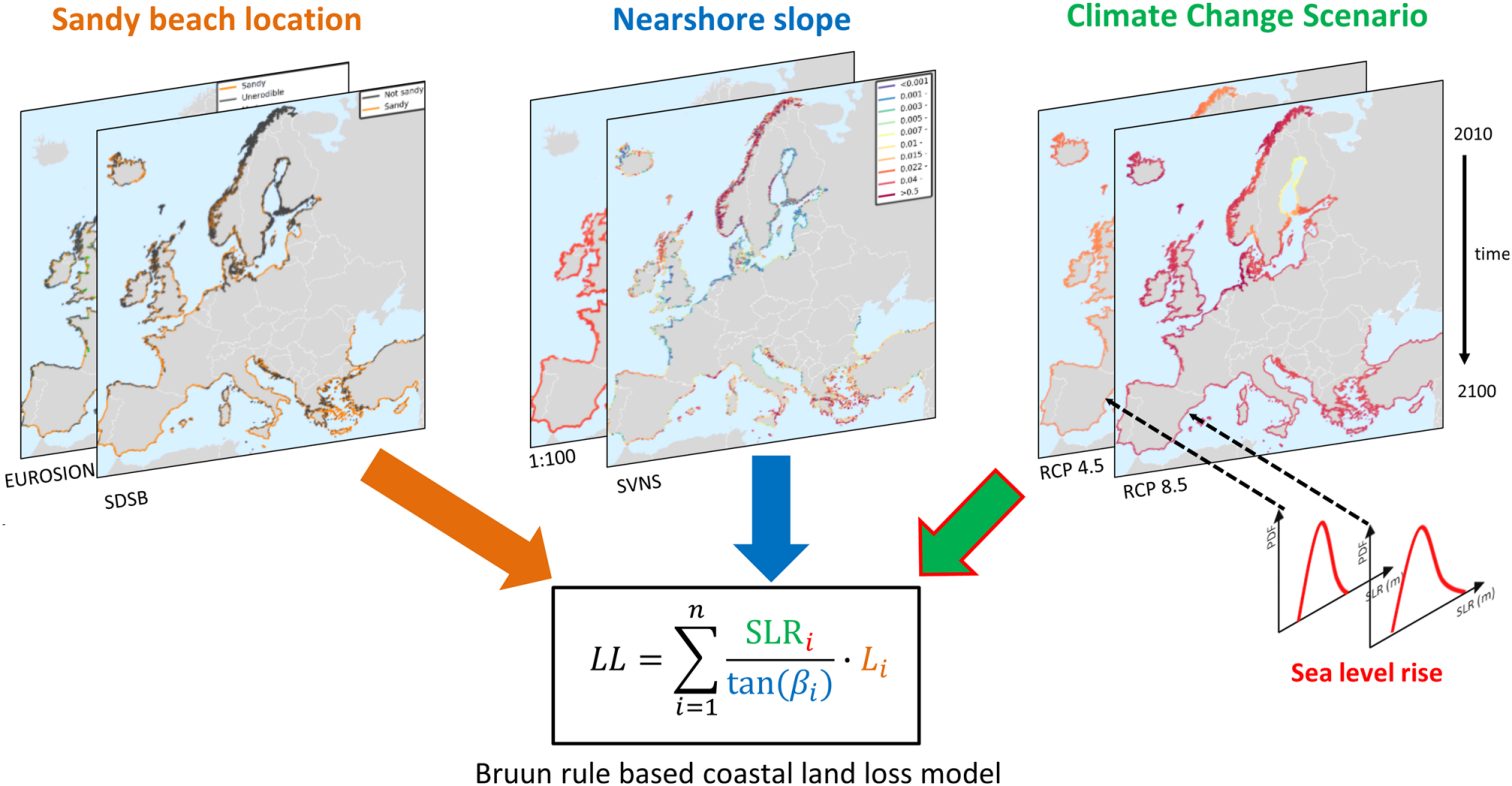
Schematization of the framework used herein to compute future European coastal land loss under the four different uncertain sources: (1) sandy beach location data, (2) nearshore slope data, (3) climate change scenario, and (4) sea level rise projections.

Our analysis is performed at an alongshore spacing of 1 km using the Open Street Maps coastline which allows us to produce an ensemble of coastal land loss projections per region and subsequently aggregate to EU Member State level. The regions used in this study are the 3^rd^ level of the Nomenclature of Territorial Units for Statistics (NUTS 3), as defined by EUROSTAT^44^. Four different geophysical data (i.e., sandy beach location and nearshore slope) combinations are generated: (a) EUROSION and 1:100 slope, (b) EUROSION and SVNS, c) SDSB and 1:100 slope and d) SDSB and SVNS. For each of these combinations the SLR driven potential shoreline retreat and coastal land loss, relative to the baseline year 2010, are estimated under RCP 4.5 and 8.5 making in total 8 different assessments. These are compared at a European and regional level. We estimate the potential shoreline retreat due to SLR using the Bruun rule and then aggregate it to coastal land loss as:

1 $$LL=\sum_{i=1}^{n}\frac{{SLR}_{i}}{tan({\beta }_{i})}\bullet {L}_{i},$$

where ${SLR}_{i}$ is the regional SLR at the time of interest, $tan({\beta }_{i})$ is the nearshore slope^41^ (see “Methods”, subsection 1 for definition) and ${L}_{i}$ is the length of each segment represented by grid point $i$ (here 1 km), while $n$ is the total number of grid points in the area of interest. The term $\frac{{SLR}_{i}}{tan({\beta }_{i})}$ is essentially the Bruun rule, which gives the potential shoreline retreat $R$ at each grid point $i$, and it is set to zero if the point is not sandy or SLR is negative. We use coastal land loss as we believe that a metric of eroded area per NUTS3 region is much more descriptive than shoreline retreat because it takes the fraction of sandy beach length over the entire cell and thus the uncertainties with respect to the sandy beach location datasets into account. Additionally, area metrics can relate to beach capacity (e.g. for tourism) and needed quantity of beach nourishment material, so overall coastal land loss can be quite relevant for stakeholders. An overview of the framework followed in the analysis herein is presented in Fig. 1.

A variance-based sensitivity analysis^25^ (see “Methods”, subsection 4) was applied to quantify the contribution of each source of uncertainty in Eq. (1) to the total uncertainty of the coastal land loss projections. These sources are (a) the dataset choice for the location of sandy beaches, (b) spatially-varying or uniform nearshore slopes, (c) the RCP scenario and d) the SLR projections uncertainty within each RCP. By assigning a probability distribution (see “Methods”, subsection 4) to each of the four aforementioned input parameters, we propagate the uncertainties of the input parameters to the coastal land loss projections, using a Monte-Carlo approach comprising 50,000 simulations per region and per decade (Supplementary Fig. S14). Uncertainty indices, describing the relative uncertainty contribution of each source defined above, are calculated for each NUTS3 region for different time horizons in the twenty-first century.

The projections thus obtained indicate the spatial variability in SLR driven potential shoreline retreat and coastal land loss and how the choice of input geophysical data (i.e., sandy beach location and nearshore slopes) can influence these projections. Additionally, the novel uncertainty analysis allows quantifying and comparing the uncertainties related to the choice of input geophysical datasets versus the inherent uncertainty associated with RCPs and SLR projections. The choice of the geophysical datasets is a type of epistemic uncertainty, meaning that it is connected with limitations in the current scientific knowledge and methods to accurately describe these characteristics of the coast at the considered spatial scale.

## Results

### Sandy beach erosion

**Figure 2:**
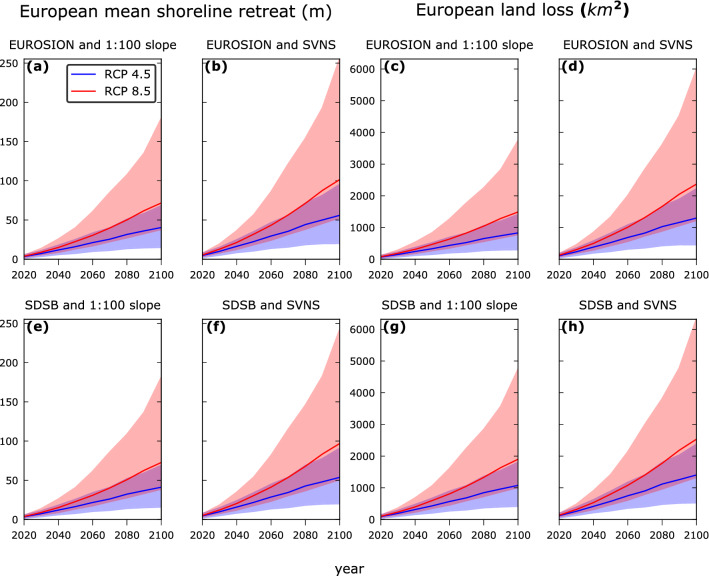
Projections of the European mean potential shoreline retreat (**a**, **b**, **e**, **f**) and European total coastal land loss (**c**, **d**, **g**, **h**) under the RCP 4.5 (blue colors) and RCP 8.5 (red colors), relative to the baseline year 2010 (in the absence of ambient shoreline changes), for the four different geophysical data (sandy beach location and nearshore slope) combinations: (**a**, **c**) EUROSION and 1:100 slope, (**b**, **d**) EUROSION and SVNS, (**e**, **g**) SDSB and 1:100 slope and (**f**, **h**) SDSB and SVNS. The solid lines are the projected potential shoreline retreat/coastal land loss under the median SLR projections, while the shaded areas indicate the 5% and 95% confidence intervals.

Using the SDSB and SVNS data, by 2050 we project a European average SLR driven potential shoreline retreat (in the absence of ambient shoreline changes) of between 18.1 and 53.9 m (5%—95% confidence interval) under RCP 8.5, relative to the baseline year 2010 (Fig. 2). At the end of the century, these projections reach values between 51 and 241.5 m. For RCP 4.5, the potential shoreline retreat by 2050 is projected to be between 10.7 and 33.9 m (5%—95% confidence interval), i.e. almost 35% smaller than that for RCP 8.5. At the end of the century, mitigation could play an even more important role with a reduction of almost 55% in average shoreline retreat under RCP 4.5 compared to that projected for RCP 8.5. The use of the EUROSION versus the SDSB dataset has a minor effect on the European average shoreline retreat, with absolute differences of less than 3.5% (Fig. 2). On the other hand, the European average shoreline retreat values computed with the SVNS data are almost 36% larger compared to that calculated with the 1:100 uniform slope assumption.

With respect to the total coastal land loss, we project 473–1,410 km^2^ (5–95% confidence interval) being lost across Europe under RCP 8.5 by 2050, using the SDSB and SVNS data (Fig. 2). By 2100 this range is projected to be between 1,334 and 6,316 km^2^. Under RCP 4.5, these values are almost 35% and 55% smaller by 2050 and 2100, respectively. The use of the EUROSION dataset instead of SDSB results in a reduction of almost 9% in the coastal land loss projections. On the other hand, when the 1:100 uniform slope assumption is used, the differences between the projections using the two different sandy beach location datasets increase, with the SDSB projecting 30% more coastal land loss.

The confidence intervals of the projections of the European average shoreline retreat and total coastal land loss (Fig. 2) per data combination are directly connected to the uncertainty in the SLR projections (Supplementary Fig. S13). These uncertainties are related to the contribution of steric SLR, ice sheets, glaciers and land–water storage^42^. At the end of the century, the uncertainty related to the dynamic behaviour of the Antarctic ice sheet dominates the total uncertainty of the SLR projections^42^, which consequently introduces a large uncertainty to the end of century shoreline retreat and coastal land loss values.

**Figure 3:**
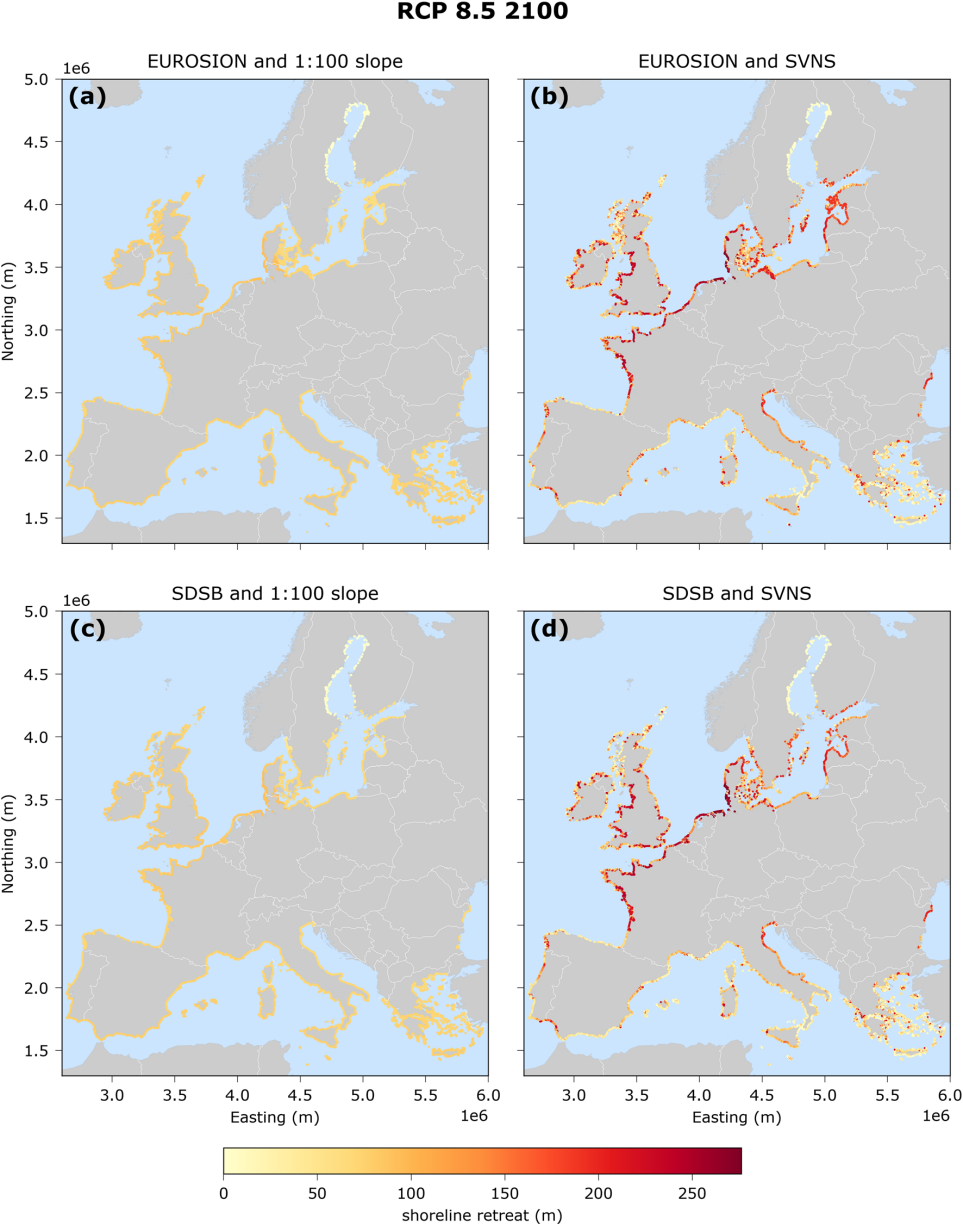
Potential shoreline retreat (m) projections at sandy beaches for the median projected SLR at 2100 under RCP8.5 (in the absence of ambient shoreline changes), relative to the baseline year 2010. Each map represents an assessment with a specific combination of geophysical data (sandy beach location and nearshore slope): (**a**) EUROSION and 1:100 slope, (**b**) EUROSION and SVNS, (**c**) SDSB and 1:100 slope and (**d**) SDSB and SVNS. The maps are projected in the ETRS89-LAEA system.

For each individual data combination, the shoreline retreat projections differ spatially across Europe (Fig. 3). As expected, when using the 1:100 uniform nearshore slope assumption, the European coastal retreat map is quite uniform with modest spatial variability which arises only due to regional SLR differences. On the other hand, when the SVNS data are used, coastal retreat across Europe is more spatially variable (Fig. 3 and Supplementary Fig. S1). Only in the North Baltic Sea is the shoreline retreat significantly low due to GIA which reduces the regional SLR in the area^43^.

**Figure 4:**
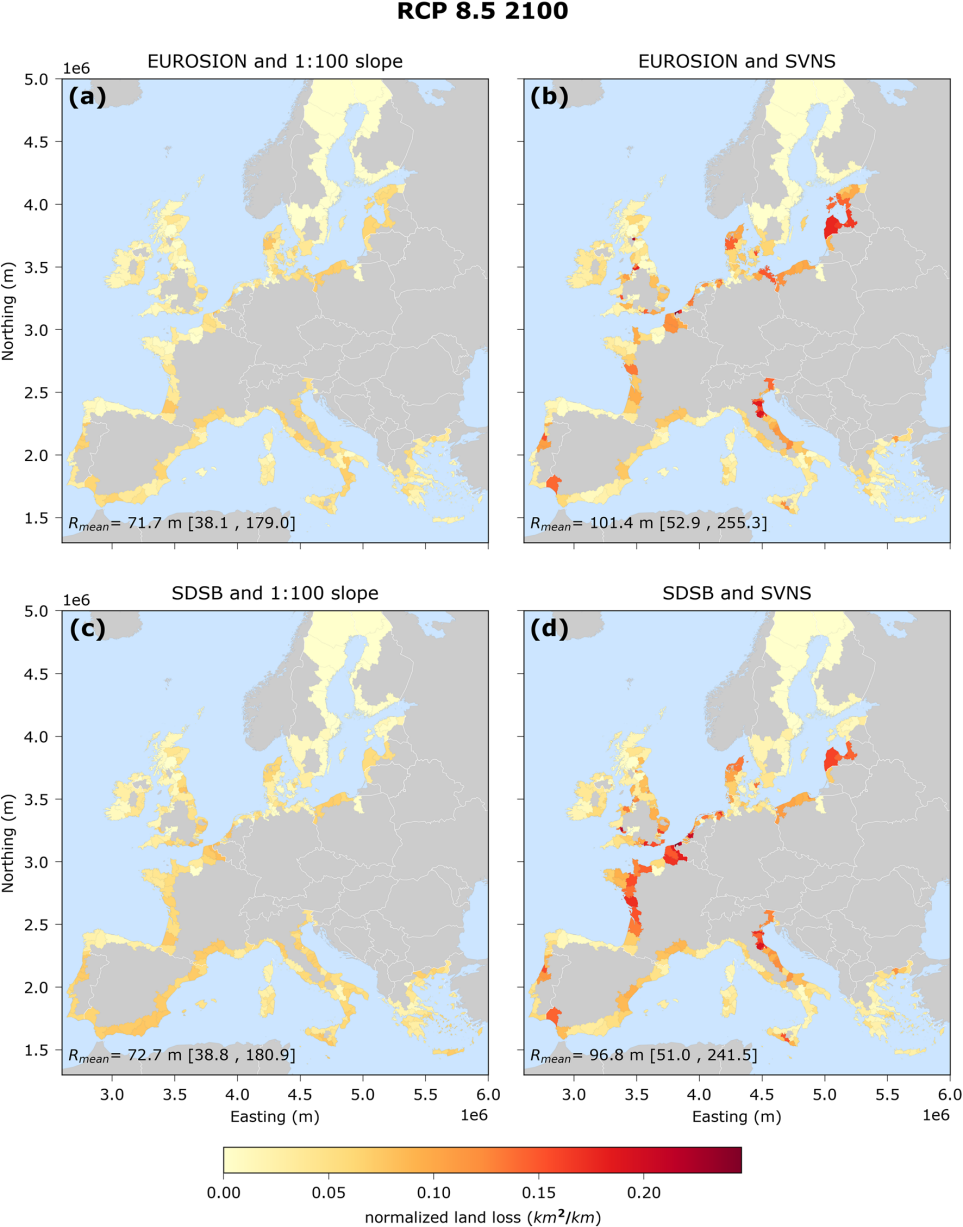
Normalized coastal land loss (km^2^/km) projections per NUTS 3 region for the median SLR at 2100 under RCP 8.5 (in the absence of ambient shoreline changes), relative to the baseline year 2010. The coastal land loss has been normalized per the coastline length of each NUTS3 region. Note that each region has a variable area and thus coastline length as defined by Eurostat. Each map represents an assessment with a specific combination of geophysical (sandy beach location and nearshore slope): (**a**) EUROSION and 1:100 slope, (**b**) EUROSION and SVNS, (**c**) SDSB and 1:100 slope and (**d**) SDSB and SVNS. The maps are projected in the ETRS89-LAEA system. The values at the bottom left of each map indicate the European average shoreline retreat (m) and are derived for median SLR projections, while in the brackets the average EU values are given for the of 5th to 95th percentiles of the SLR projections.

The coastal retreat values calculated per location are aggregated to coastal land loss at the NUTS3 regional level using Eq. (1), and then normalized according to the coastal length of each NUTS3 region. This results in maps that indicate vulnerable NUTS3 regions which could potentially lose large coastal areas with respect to their total coastal length. The spatial distribution of the normalized coastal land loss per region (in the absence of ambient shoreline changes) varies between the four data combinations but has a similar spatial pattern for RCP 8.5 (Fig. 4 and Supplementary Fig. S3) and RCP 4.5 (Supplementary Figs S2 and S4), with the magnitude differing significantly. The normalized coastal land loss (km^2^ of area lost / km of coastline) is the combination of the coastal retreat per location, which is in turn connected to the SLR and the average nearshore slopes, and the relative length of the sandy coastline. When using the SVNS data and irrespective of the sandy beach distributions dataset, highly vulnerable regions are identified on the Italian Adriatic coast, the French Atlantic coast, Belgium, The Netherlands, Denmark, Lithuania and Latvia (Fig. 4).

### Uncertainty analysis

**Figure 5:**
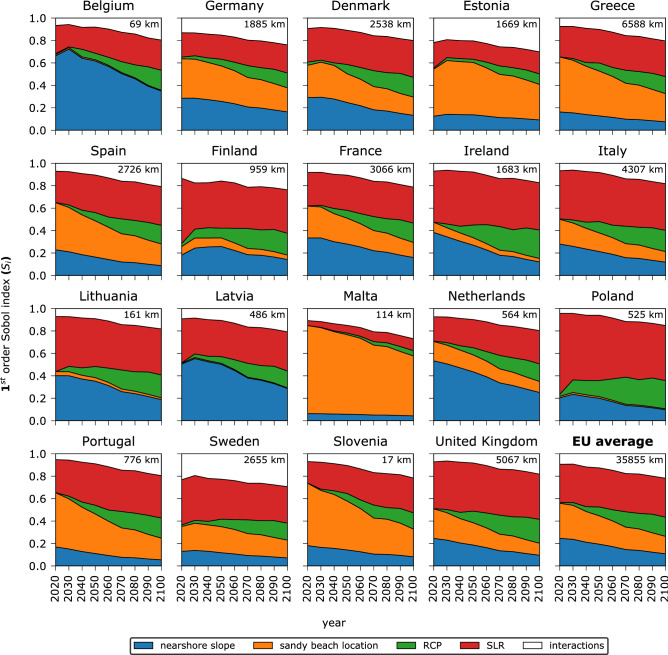
Variance-based global sensitivity analysis of the coastal land loss model as a function of time for different EU countries. For each year considered, the width of each patch indicates the fraction of the variance of the coastal land loss projections (in the absence of ambient shoreline changes) that could be removed if the respective input parameter was known. White areas indicate interactions between parameters. The 1st order Sobol indices presented here are calculated as the weighted average of the country’s regions (with respect to the sandy coastline length). The total potential sandy coastline length (EUROSION or SDSB) is indicated at the top right corner of each graph. On the bottom right an EU average plot shows the average values over all of Europe.

As the results presented above indicate, the choice of the input geophysical datasets (i.e., sandy coastline and nearshore slope) is critical for the representation of the coastal retreat along the European coastline. The relative contribution of the different sources of uncertainty to the total uncertainty (being equal to unity in every year) and their temporal evolution per European country are quantified using a global sensitivity analysis (Fig. 5). The main effect of each source of uncertainty in Eq. (1) to the total variance of the coastal land loss projections (in the absence of ambient shoreline changes) per NUTS3 region is described by the 1st order Sobol index (see “Methods”, subsection 4). This value can be interpreted as the relative amount of the total variance that would be removed if the true value of the parameter was known^25^. The global sensitivity analysis was performed for each NUTS 3 region, mapping the contribution of each parameter to the total variance through the twenty-first century and then aggregated per country. The temporal evolution of each source through the century differs significantly among countries (Fig. 5). As expected, an increase of the uncertainty due to the selected RCP is observed towards the end of the century for all countries, as indicated by the green shaded area in Fig. 5. Even though sandy beach location and nearshore slope information is constant in time, their Sobol indices can be time dependent, since Sobol indices describe the relative contribution of each input parameter to the total variance. For the same reason, the uncertainty contribution of SLR projections can be high at the beginning of the century for some countries, because even though the variance of the SLR projections is small, it can have large relative effect, since it is the main driving force of changes in the coastal land loss model.

Figure 5 also indicates that European countries can be divided into three groups with respect to the different uncertainty contributions. There are countries (Poland, Ireland, Italy, United Kingdom, Lithuania, Finland, Sweden and Denmark representing 50% of total sandy coastline) where the relative contribution of the variance related to the climate change scenario and the SLR projections dominate by the middle of the century (72.6%, 58.6%, 53.9%, 53.3%, 52.5%, 50.7%, 42.3% and 38.8% by 2050 respectively). This type of uncertainty is inherent and is connected to unknowns in future climate change mitigation policies and physical processes as e.g., ice sheet dynamics. A second group (Malta, Estonia, Slovenia, Greece, Portugal, Spain and Germany representing 38% of total sandy coastline) have a quite high relative contribution to the total uncertainty due to the choice of the sandy beach distribution dataset (70.9%, 46.7%, 43.5%, 39.9%, 35.3%, 32.3% and 31.6% by 2050 respectively). This is an artefact of a regional disagreement between the total sandy coast length of the two datasets considered here, which overshadows the uncertainty of the other parameters. In the third group (Belgium, Latvia, Netherlands and France representing 12% of total sandy coastline) the variability of coastal land loss projection is dominated by the nearshore slope uncertainty (61.7%, 50.2%, 43.5% and 28.0% by 2050 respectively). In these countries the largest contribution to coastal land loss variance is from the difference between the average nearshore slopes as calculated by the SVNS dataset and the commonly used assumption of 1:100.

The uncertainty of the coastal land loss projections could be reduced on average by up to 45% in 2050 and by 26% by 2100 if the exact spatial distribution of sandy beaches across Europe and their nearshore slopes were known (Fig. 5). Still, these values can vary significantly when considering specific regions (Supplementary Figs S5 and S6), as agreement between datasets and differences in SLR projections can vary locally. Note that the white area in Fig. 5, which for most of the countries is smaller than 20% by 2100, indicates the interactions between the parameters meaning that the combined variation of the considered parameters also contributes to the total variance. This for example can be related to the spatial dependency of the variables used herein, since for each of the sandy beach location datasets, different combinations of SLR projections and nearshore slopes will be used.

## Discussion

Our results provide novel insights with respect to the use of different geophysical datasets and assumptions in large scale assessments of SLR driven shoreline retreat and consequent coastal land loss. The use of the SVNS dataset instead of the uniform slope assumption resulted in an increased estimate of European average shoreline retreat. This is due to the fact that the SVNS based slopes are on average milder than 1:100 across Europe^41^. On the other hand, the differences in the European coastal land loss predictions (Fig. 2) are connected to differences in the sandy beach distribution and total length of sandy beaches in the EUROSION and SDSB datasets. Their point-to-point agreement was 36% along the total European coastline (Supplementary Fig. S10). The agreement was larger than 50% in most NUTS3 regions, with the exception of the Atlantic coast of Spain, some regions in France and the UK, Sweden, Finland and Greece (Supplementary Fig. S11).

The European average shoreline retreat due to SLR calculated herein with the SDSB and SVNS datasets compares well with the results from a previous global assessment that used the same input, but with some overestimation related to the use of a Bruun Rule correction factor in that study^31^, which is described by a triangular probability distribution ranging from 0 to 1 with a peak at 0.7. An older study at the European scale by Hinkel et al.^29^ which employs the assumption of uniform 1:100 nearshore slopes, projected a total coastal land loss of about 450 km^2^ (300 km^2^) by 2100 under IPCC SRES A2 (B1). The projected median coastal land loss when using the 1:100 uniform slopes here is about 1,700 km^2^ (950 km^2^) under RCP 8.5 (4.5). These results are not directly comparable since they are based on different greenhouse gases concentration pathways, but differences can be attributed to a) mainly the use of different mitigation scenarios, SLR projections and baseline years, b) differences in the coastline definition and thus coastline length, and c) sandy beach distribution differences between their study and the assessment presented herein.

The present analysis has inherent limitations and assumptions. Despite its widespread use, the Bruun Rule has been criticized^45,46^ for its assumptions and simplifications. These include: (a) that an equilibrium profile is always attained, (b) that there is a cross-shore sediment balance (no sediment sinks and sources) and (c) that it does not take into account alongshore sediment transport gradients. Additionally, recent studies have shown that the Bruun rule tends to overestimate coastal retreat when compared to physics-based probabilistic numerical models^17,19,25^. This could be potentially added as a correction factor in Eq. (1) in future studies (similarly to Vousdoukas et al.^31^) and modelled as a stochastic variable in the uncertainty analysis. The use of a physics-based model was not feasible within the scope of the present study due to time constraints as a result of the regional spatial scale of the assessment. The contribution of the choice of the coastal impact model has been previously shown to account for 20–40% of the variance in shoreline change projections by 2100 in various sites in France^25^.

**Figure 6:**
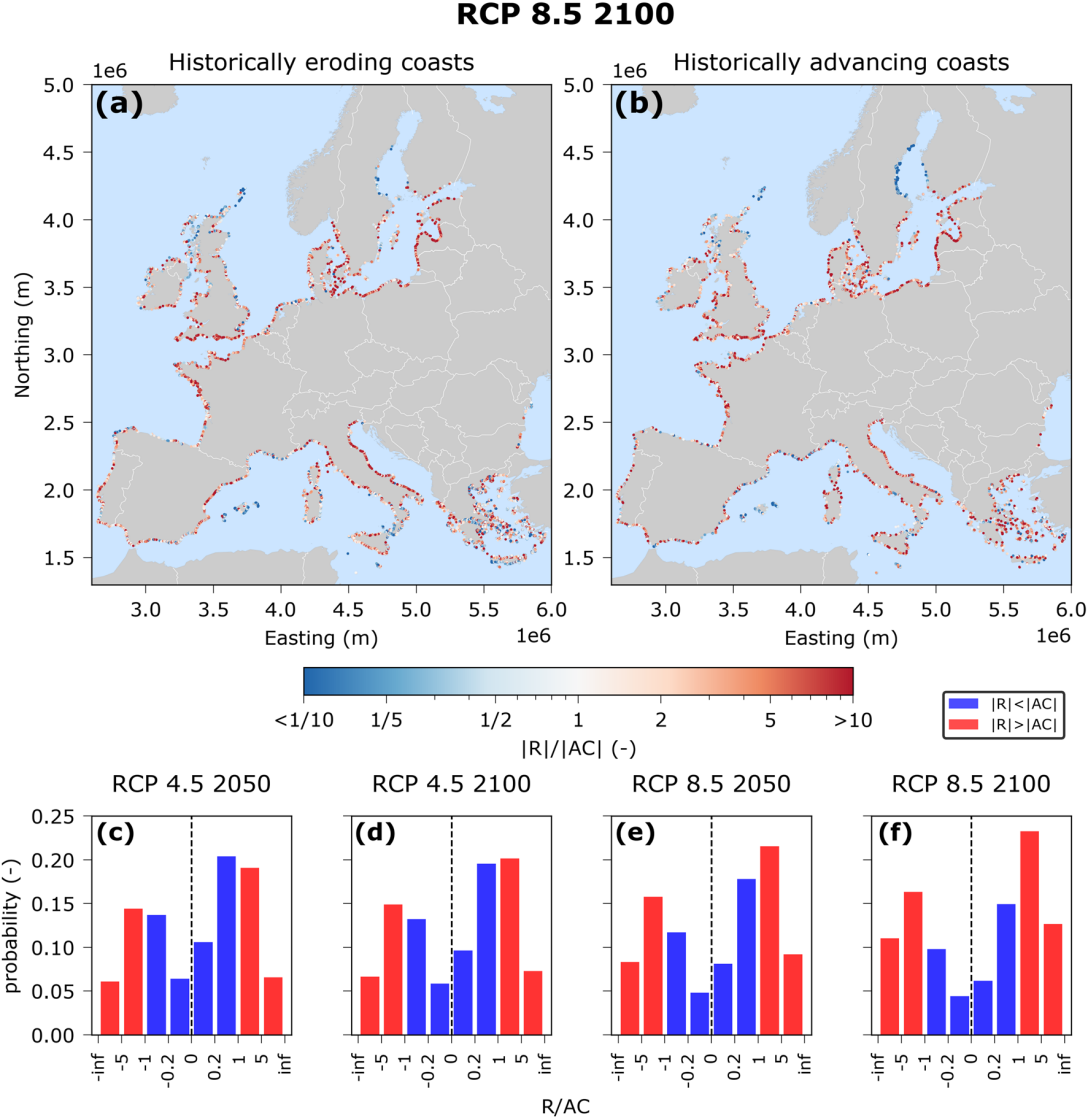
Ratios of SLR driven shoreline retreat to erosion due to ambient shoreline changes |R|/|AC| for median projected SLR at 2100 under RCP8.5, for historically eroding (HE) coasts (**a**) and historically advancing (HA) coasts (**b**). Histograms of ratios R/LT for historically eroding (R/AC > 0) and historically advancing (R/AC < 0) coasts for RCP 4.5 (**c**, **d**) and RCP 8.5 (**e**, **f**). For the comparison the points from Vousdoukas et al. 2020 were interpolated to the points of the present study by using inverse distance interpolation at a 2 km radius.

Shoreline change is a dynamic process that is not only dictated by SLR. Long-term changes in shoreline position can be expected in response to long-shore sediment transport gradients^22,31,47^. Additionally, the presence of sediment sources/sinks^37,48^ and any residual effects of storms and others seasonal, annual or multi-annual fluctuations^49^ can lead to additional shoreline changes^22,25,31,37,48^. In the work herein, we focused on the shoreline retreat that is directly driven by SLR, omitting the uncertainty related to the aforementioned additional processes. Therefore, to investigate whether the SLR-induced shoreline retreat calculated herein is the dominant mechanism of future shoreline retreat, we compare our results with projections of historic shoreline changes^31^. These ambient changes are derived from historical change rates of shoreline position based on publicly available satellite imagery^9,39^ assuming that these trends can be extrapolated into the future. We therefore calculate the ratio of median SLR induced shoreline retreat ($R$) to the median long-term ambient changes ($AC$) for each sandy location along the European coastline, differentiating between historically eroding (i.e., $AC>0$) and historically advancing (i.e., $AC<0$) coasts (Fig. 6 and Supplementary Fig. S12). We then distinguish between two groups; SLR dominated coasts ($\left|R\right|>\left|AC\right|$) and ambient changes dominated coasts ($\left|R\right|<\left|AC\right|$). We find that under RCP 4.5, $AC$ is slightly more important than $R$ in 2050, while in 2100 their relative importance is quite similar for both historically eroding and advancing coasts. Under RCP 8.5 though, in 2050, $R$ is more important than $AC$ over Europe, while in 2100 the dominance of $R$ over $AC$ becomes even more pronounced. These insights show that at least under RCP 4.5 ambient shoreline changes can be of the same importance as SLR driven shoreline retreat overall in Europe. On the other hand, under RCP 8.5 SLR driven shoreline retreat dominates especially at the end of the century when SLR rates increase. Historically advancing coasts represent almost 40% of the sandy coastline studied herein. Up to 14% of the sandy coastline can have an overall land gain by 2100 and under RCP 8.5 if the accreting historic trends continue ($|R|<|AC|$ and $AC<0$). For these coasts (e.g. heavily nourished beaches along the Dutch coast), SLR driven shoreline retreat could potentially be counteracted with continuing interventions at the historic rate under RCP 4.5. Obviously, there are distinct spatial patterns (Fig. 6) which show quite some spatial variability of the ratio. Furthermore, the assumption of the long-term shoreline change rates to remain constant in the future is also somewhat tenuous.

For the sake of completeness, the projected shoreline retreat induced by SLR alone ($R$) were compared with projections of total potential shoreline retreat ($dx$) that includes the aforementioned ambient shoreline change ($AC$). Here, $dx$ was estimated in a probabilistic manner, by sampling $R$ and $AC$ from their distributions in a Monte Carlo simulation and adding them up to create probabilistic $dx$ projections for 2050 and 2100 under RCP 4.5 and 8.5. As an illustrative example, European maps of the median dx values across Europe thus obtained are shown in Supplementary Fig. S15, indicating that the general spatial patterns when $AC$ is included stay the same for the most part, with the exception of a few locations where a shoreline advance is projected (e.g., Dutch coast and North Baltic Sea) or where more severe shoreline retreat (than R alone) is projected. The variance of the projected distributions of $dx$ is somewhat larger than that of the variance associated with projections of $R$ alone (Supplementary Fig. S1).

Other missing uncertainty factors include the choice of the parameter distributions used^25^ in the global sensitivity analysis, which can affect the total variance of coastal land loss projections and subsequently alter the relative contribution of each variable. For example, assigning the same probability to the choice of the two sandy beach location datasets and the two nearshore slope approaches is a choice that directly affects the uncertainty quantification. For the former, this choice derives from the spatial extent of the dataset which makes it difficult to assign different performance levels for the two datasets. For the latter, even though we can expect that the SVNS dataset will contribute to better estimations of shoreline retreat than the assumption of a uniform slope, we assign the same probability to quantify the sensitivity of this choice and to be able to compare our results with previous studies^29,30^. Additionally, we treat the geophysical characteristics of the coastline (i.e. location of sandy beaches and nearshore slopes) as constants in the future and thus described by the current available datasets, even though at the end of the century changes in hydrodynamics and sediment supply might alter them.

Our uncertainty analysis is based on choices with respect to the geophysical representation of the coastline that need to be made in large-scale shoreline retreat assessments. For this reason, we use available large-scale datasets that cover all of Europe or a large part of it (see “Methods”, subsection 2 for spatial coverage of EUROSION). Uncertainty related to geophysical representation is epistemic and is mainly due to the availability, spatial coverage and accuracy of data and observations. However, at the regional or country scale, better resolution and higher accuracy data are seldom available. Exceptions are The Netherlands and Belgium where the relatively smaller coastline length and long-term surveying have contributed to the creation of detailed coastal bathymetric datasets available at country level^50^. Where such data are available, the uncertainty arising from the sandy beach location and nearshore slopes can be reduced significantly. Thus, data acquisition and observational programs could provide better representation of the coastal system and reduce the uncertainties in shoreline retreat projections, especially for the 2nd and 3rd group of countries described in Results subsection 2. Conversely, uncertainty related to future SLR projections (1st group of countries described in Results subsection 2) is more difficult to reduce, since it is related to global carbon emission reductions, economic developments, and inherit uncertainties in the physical processes that will lead to SLR.

The SLR projections used here include the contribution from GIA but not the local tectonics and subsidence effects^42^. Thus, the relative sea level rise in areas with significant subsidence (e.g. Po Delta in Italy^51^) can be larger than the values used here. Nevertheless, there is to date no Europe-wide map of subsidence projections available. Additionally, due to the effect of GIA, there are regions (mainly in North Baltic Sea) where there is negative SLR, and for which we assume a zero coastal retreat in the study herein as the Bruun rule has in fact not been derived to explicitly account for coastal accretion due to sea level drop. Nevertheless, we expect that negative SLR would contribute to land gain in these areas (which roughly account for almost 3% of the European transects).

Regional coastal land loss projections presented here are sensitive to the spatial extent of the regions used, due to the spatial characteristics of the input datasets. This means that the level at which the coastal retreat per segment is aggregated to coastal land loss is important for both the (normalized) coastal land loss projections and the uncertainty analysis. Here we used the NUTS 3 level, as it is the smallest administrative unit defined from the European Union available and we believe that at that level our results could be most useful for policy making and adaptation planning. Furthermore, our projections of shoreline retreat of sandy coastlines assume that a sufficiently wide erodible beach is present. However, it should be noted that at some locations the erodible beach width could be smaller than the projected retreat, leading to an overestimation of coastal land loss therein. Nevertheless, as observations of beach width at the European scale are not readily available, the erodible beach width cannot be taken into account at present. That is why our projections are defined as potential shoreline retreat and coastal land loss. When European-wide information becomes available on the actual spatial distribution of sandy beach width our results could be translated to actual beach loss.

## Conclusions

The present analysis focused on shoreline retreat and regional coastal land loss projections at the European scale driven only by sea level rise (SLR), in the absence of any other natural processes or human influences that could result in shoreline change. Against previous studies at this scale, here we employ different geophysical datasets, identifying the sensitivity of this choice. The spatially averaged median value of SLR driven potential shoreline retreat of sandy beaches in Europe by 2100, relative to the baseline year 2010, is projected to be about 97 m under RCP 8.5 and 54 m under RCP 4.5. This translates to a potential coastal land loss of about 2,500 km^2^ under RCP 8.5 and 1,400 km^2^ under RCP 4.5 for the same time period along the European coastal zone. For coastlines with historically advancing trends larger than that of the SLR retreat ones (about 14% of the European coastline studied herein), overall net changes on coastal land area might result in land gain. The use of spatially-variable, data-based nearshore slope information identified a number of coastal land loss hotspots around Europe and higher shoreline retreat values compared to projections provided by previous assessments that used the assumption of a uniform 1:100 slope throughout Europe. These hotspots include regions along the Italian Adriatic coast, the French Atlantic coast, the east part of the Baltic Sea and around the North Sea.

An analysis of the temporal evolution of the composition of the total uncertainty associated with projected SLR driven coastal land losses indicated that the choice of the input geophysical datasets (i.e. sandy beach location and nearshore slope) is an important source of uncertainty for a large number of European coastal regions especially until 2050. By the end of the twenty-first century, the uncertainties associated with climate change scenario and SLR projections become more dominant. On average, the variance of coastal land loss projections could diminish by almost 45% in 2050 and 26% in 2100 if accurate data on sandy beach location and nearshore slope were used. In Malta, Estonia, Slovenia, Greece, Portugal, Spain and Germany, the choice of the sandy beach location dataset was the most important source of uncertainty, while for Belgium, Latvia, Netherlands and France the choice of spatially varying versus uniform slopes was the most important choice. For the rest of European countries, the uncertainties associated with unknown future scenarios and the SLR projections dominated the total uncertainty in projections.

## Methods

### Coastal impact model

We used the Bruun rule^34^ to quantify the direct erosion of sandy beaches due to SLR. This approach is based on a simple two-dimensional mass conservation rule assuming that an equilibrium profile will be preserved in the future when SLR occurs. It employs a number of assumptions and simplifications (i.e. equilibrium profile existence, cross-shore sediment balance), that have made it a controversial matter, debated in the literature for decades^45,46^. Nevertheless, it is, at present, the only method that can produce coastal recession estimates due to SLR at large spatial scales in a computationally efficient manner. The Bruun Rule is expressed as:

2 $$R=\frac{SLR}{tan(\beta )},$$

where $R$ is the horizontal shoreline retreat, SLR is the sea level rise, and $\beta$ is the active profile slope. As active profile slope here we describe the slope between the depth of closure (i.e., the depth beyond which observed bed level changes show no significant changes in time) and the shoreline^34^ which we call herein nearshore slope. We also assume that if SLR is negative there will be no shoreline retreat.

The European coastline was discretized with an alongshore spacing of 1 km to have a good coverage of the alongshore variability of the coastal profiles, while keeping the computational costs at a feasible level. The coastline was defined by the Open Street Maps dataset of 2016^52^. This approach has been previously used for a global assessment of historical coastal erosion^9^. Our projections coverage is determined by the overlap of the two sandy beach distribution datasets used (see next section). This was defined by the EUROSION dataset which includes the coastal EU counties except Croatia, and Romania, Bulgaria and Cyprus which are partially covered.

### Geophysical data

The spatial distribution of sandy beaches at the European scale is a critical input for assessing shoreline retreat. Here we used two datasets that describe the location of sandy beaches and have European-scale coverage. The first was the geomorphological map of the European coasts available from the EUROSION project^40^. This map includes 20 different classes and it is available at a coastal segment level with coherent characteristics (e.g. geomorphology, geology, erosion rates and more) and various lengths. These data were extracted as points with a 250 m alongshore resolution, using GIS. The resulting data-points were used to populate the alongshore point grid described in the previous section, employing proximity analysis. Another coastal geomorphological map is available for the Mediterranean^53^, which classifies coastal segments in four classes and was used herein to reclassify the EUROSION dataset. The classes that were used are (a) sandy beaches, (b) unerodable coasts, (c) muddy coastlines and (d) rocky coasts with pocket beaches. The EUROSION classes were then regrouped in these four classes according to the highest occurrence as described in Athanasiou et al.^41^. Ultimately, only the alongshore points that were classified as sandy beaches were used for the assessment of future shoreline retreat and coastal land loss. For the points classified as “rocky with pocket beaches” a relative percentage of sandy beach length per 1 km long segments was retained using the length of pocket beaches following Monioudi et al.^26^.

The second dataset was the Satellite Derived Sandy Beaches (SDSB) location dataset^9^. This dataset has a global coverage and was created using machine learning techniques on satellite images from the Sentinel-2 mission. It classifies locations with an alongshore spacing of 500 m as sandy or not sandy. Using the same method as for the EUROSION data, the alongshore points of our computational vector were populated with the SDSB data, creating another layer of sandy beaches location (Supplementary Fig. S7).

The coastal recession computed using the Bruun rule is linearly dependent on nearshore slope (Eq. 2). A recent study produced a global dataset of nearshore slopes, using global topo-bathymetric data at a 1 km alongshore resolution^41^ (called SVNS dataset herein). The spatial variability of $\beta$ as indicated from this dataset is quite distinctive in Europe (Supplementary Fig. S8). In order to compare the importance of the spatial variability of the nearshore slope against the so far commonly used assumption of a uniform slope of 1:100, we used both approaches when applying Eq. (1). To constrain nearshore slopes to the naturally encountered values we used an upper and lower limit of 1:5 and 1:300^31^. The points that had values out of this range were constrained to the limits. This was necessary to take into account outliers from the SVNS dataset and consider that in some cases the sandy beach location datasets can erroneously indicate presence of sandy beaches in non-sandy coastlines. From the sandy segments across the European coastline extents that were considered presently, we found that about 51.5% have slopes milder than 1:100 (as defined between the depth of closure and the shoreline).

### Sea level rise data

We used the results of a global probabilistic, process based study^4,42^ to obtain SLR projections around Europe up to end of the twenty-first century. The data were available at offshore points with a spacing of about 50 km. Projections were available from 2010 to 2100, every 10 years, capturing the 5th, 17th, 50th, 83th, 95th and 99th percentiles of potential SLR under RCP 4.5 and RCP 8.5. For RCP 8.5 we use the high-end RCP 8.5 scenario from Jackson and Jevrejeva^42^ which is based on IPCC AR5^2^ but uses Antarctic and Greenland ice sheet contributions from Bamber and Aspinall^54^, resulting in higher median global SLR (i.e., 84 cm versus 74 cm of AR5) and larger and more asymmetric uncertainties^4^. These projections were of regional SLR, thus taking into account the regional footprint of SLR, due to various contributions as described in Jackson and Jevrejeva^42^. On the other hand, local tectonics and subsidence are not included in the projections. For each computational point a specific SLR value was attributed for each decade, RCP and percentile using proximity analysis (Supplementary Fig. S9). Then an empirical cumulative distribution function (ECDF) was created for each location and RCP scenario, using the percentile values.

### Uncertainty analysis

A variance-based sensitivity analysis was used to quantify the uncertainty of the coastal land loss estimates produced using Eq. (1). This method measures the contribution of each of the uncertain input parameters $Xi$ to the variance of the outcome of a model $Y$ ^55,56^ and has been previously applied in various studies of coastal impacts^25,57,58^. On this basis, the 1st order Sobol indices, which range between zero and one, can be defined as follows:

3 $${S}_{i}=\frac{Va{r}_{{X}_{i}}\left({E}_{{X}_{\sim i}}\left[Y|{X}_{i}\right]\right)}{Var\left(Y\right)}$$

where ${\rm X}_{\sim i}$ denotes the matrix of all parameters but ${X}_{i}$. The first-order indices ${S}_{i}$ can be interpreted as the main effects of ${X}_{i}$, i.e. the proportion of the total variance of the output $Y$ that would be removed if we knew the true value of parameter ${X}_{i}$. This index provides a measure of importance (i.e. a sensitivity measure) useful when ranking, in terms of importance, the different input parameters^55^.

We applied the variance-based sensitivity analysis on Eq. (1), using as inputs: the choice of the 2 geophysical datasets (i.e., sandy beach location and nearshore slopes), the unknown mitigation scenarios (i.e., RCP 4.5 or RCP 8.5) and the SLR projections (Fig. 1). A discrete uniform distribution with the same probability of 50% was applied for the choices of geophysical datasets and the RCPs, assuming that there is no information on which one performs better (for the geophysical datasets) or which is most probable to occur (for the RCPs). Sea level rise uncertainty was described by the probability distribution for each location and decade, per RCP as described in the previous subsection.

For each NUTS3 region and decade, auxiliary random variables uniformly distributed in [0,1] were sampled for each parameter, following Le Cozannet et al.^58^. Then the inverse cumulative distribution functions of each parameter were used to translate these to the actual parameters. Each of the four input parameters was sampled from the aforementioned distributions following a quasi-random Sobol sequence of 5,000 samples, resulting in 50,000 input parameter combinations which were propagated through Eq. (1) using a Monte Carlo approach, leading to 50,000 realizations of coastal land loss per NUTS3 region and per decade. The sampling of the SLR was performed from the specific SLR distributions of each region and decade. An outline of the framework can be seen in Supplementary Fig. S14. The number of samples was chosen in order to achieve a precision of the Sobol indices in the order of 2% while maintaining computational costs within reasonable limits. The Python package SALib was used to perform the analysis^56,59^.

## Supplementary information

Supplementary figures.

## Data Availability

The projections of shoreline retreat are available at https://doi.org/10.4121/uuid:8e73cab0-960b-46a8-bf67-ee0eadcc1e7d.
