# Supplementary material for: Uncertainties in projections of sandy beach erosion due to sea level rise: an analysis at the European scale
Source: Sci Rep. 2020 Jul 17;10:11895. doi: 10.1038/s41598-020-68576-0 (PMC7367847; doi:10.1038/s41598-020-68576-0)
Supplement: Supplementary file 1 — Supplementary figures. [file 41598_2020_68576_MOESM1_ESM.pdf]

Supplementary Information

Uncertainties in projections of sandy beach loss due to sea level rise: an analysis at the European scale

Panagiotis Athanasiou[1,2*], Ap van Dongeren[1,4], Alessio Giardino[1], Michalis I. Vousdoukas[3], Roshanka Ranasinghe[4,2, 1], Jaap Kwadijk[2,1]

[1] Deltares, PO Box 177, 2600 MH Delft, the Netherlands

[2] Water Engineering and Management, Faculty of Engineering Technology, University of Twente, PO, Box 217, 7500 AE Enschede, the Netherlands

[3] European Commission, Joint Research Centre (JRC), Via Enrico Fermi 2749, I-21027, Ispra, Italy

[4] Department of Water Science and Engineering, IHE Delft Institute for Water Education, PO Box 3015, 2610 DA Delft, the Netherlands

**Correspondence to*:

Panagiotis Athanasiou

Deltares, P.O. Box 177, 2600 MH Delft, The Netherlands

Email: panos.athanasiou@deltares.nl

Tel.: +31 650000860

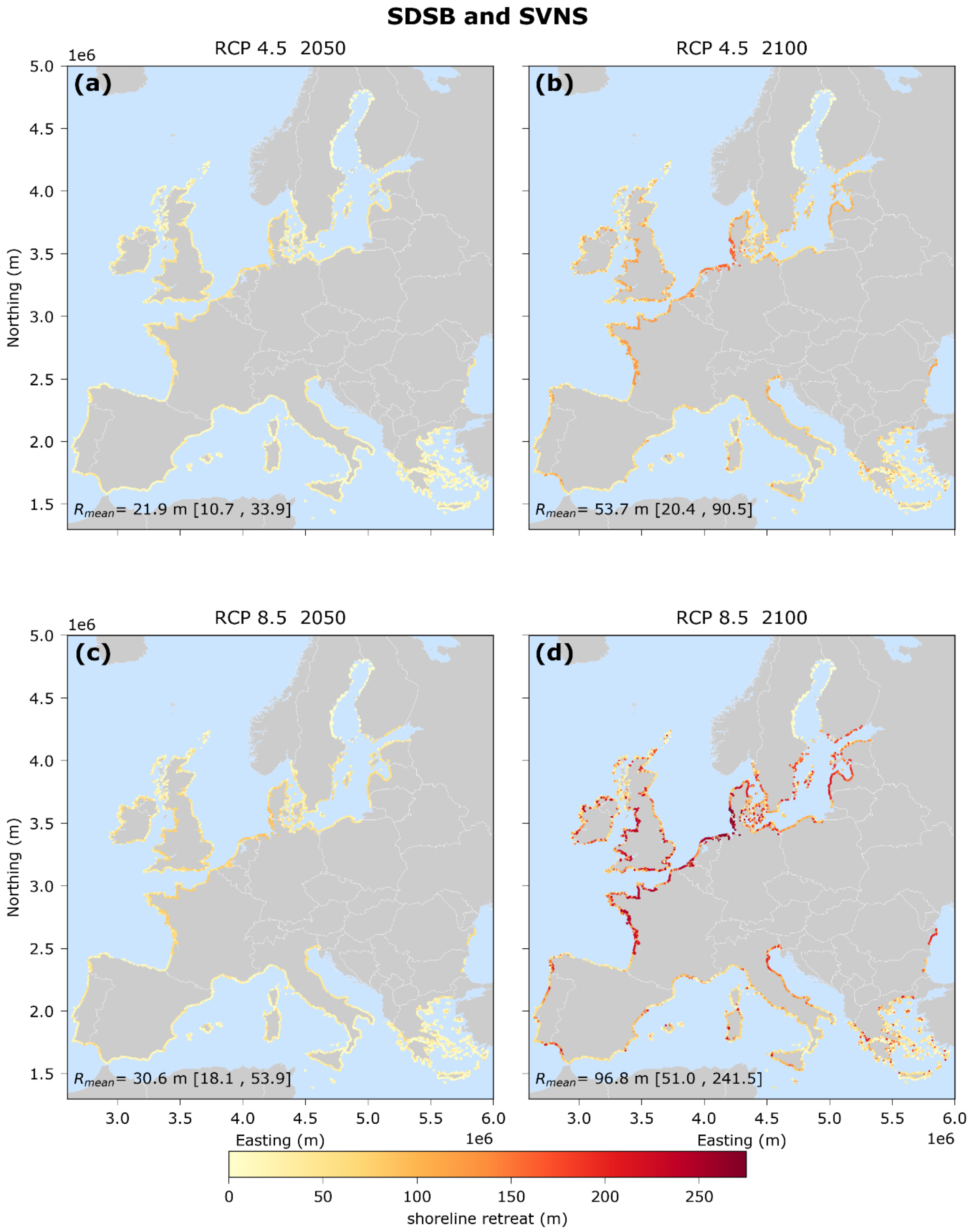

Figure S.1: Potential shoreline retreat (m) projections at sandy points for the median SLR under RCP 8.5 (c,d) and RCP 4.5 (a,b) at 2050 (a,c) and 2100 (b,d), relative to the baseline year 2010 (in the absence of ambient shoreline changes). The maps represent an assessment using the SDSB sandy beach distribution dataset and the SVNS slopes data. The values at the bottom left of each map indicate the European average shoreline retreat (m) and are derived for median SLR projections, while in the brackets the mean EU values are given for the of 5th to 95th percentiles of the SLR projections. The maps are projected in the ETRS89-LAEA system and were created with Python 3.7.3 (https://www.python.org) using the Geopandas (v0.6.2, https://geopandas.org/) and Matplotlib (v3.1.2, https://matplotlib.org/) libraries.

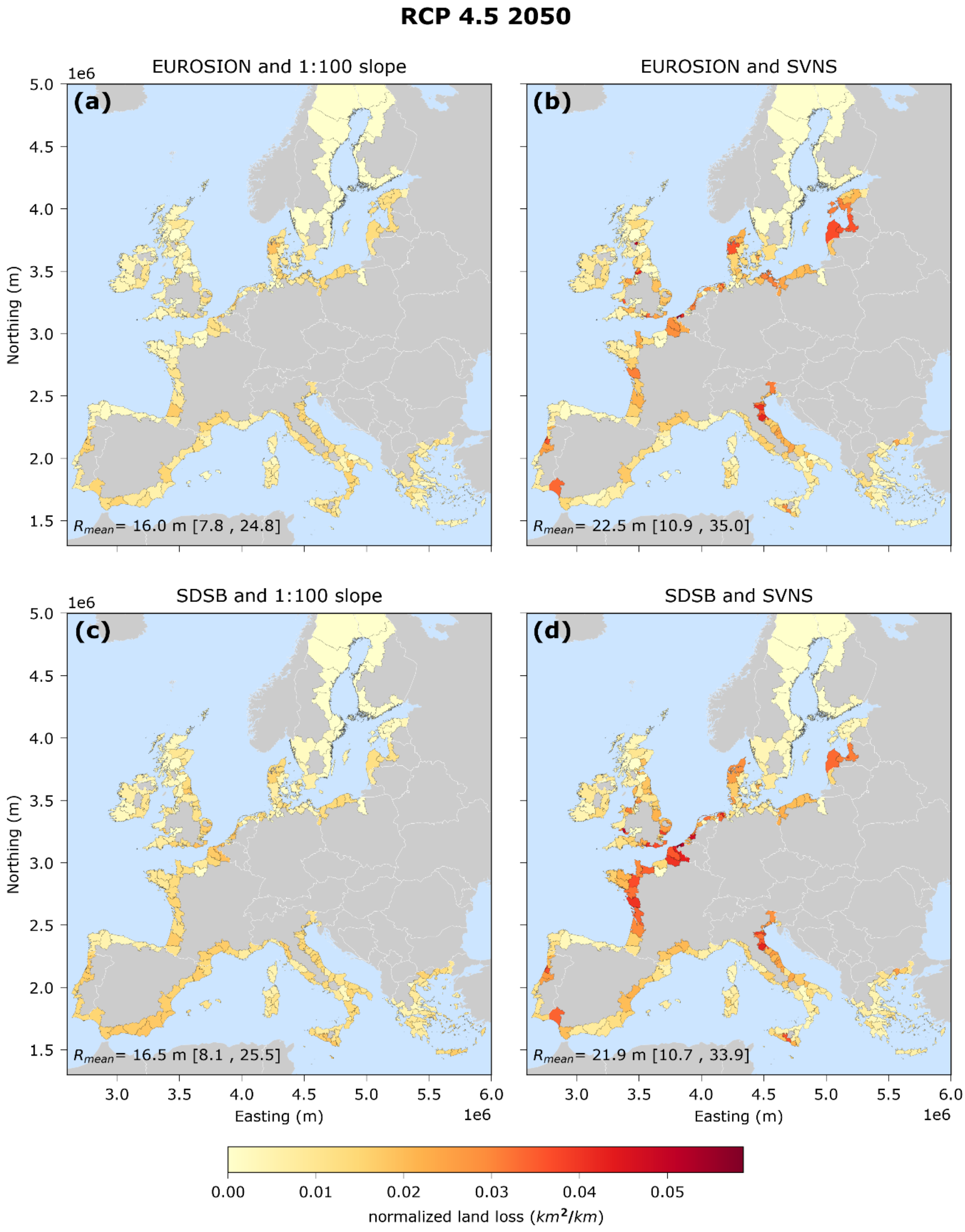

Figure S.2: Normalized coastal land loss (km²/km) projections per NUTS 3 region for the median SLR at 2050 under RCP 4.5 (in the absence of ambient shoreline changes), relative to the baseline year 2010. The coastal land loss has been normalized per the coastline length of each NUTS3 region. Note that each region has a variable area and thus coastline length as defined by Eurostat. Each map represents an assessment with a specific combination of geophysical (sandy beach location and nearshore slope): (a) EUROSION and 1:100 slope, (b) EUROSION and SVNS, (c) SDSB and 1:100 slope and (d) SDSB and SVNS. The values at the bottom left of each map indicate the European average shoreline retreat (m) and are derived for median SLR projections, while in the brackets the mean EU values are given for the of 5th to 95th percentiles of the SLR projections. The maps are projected in the ETRS89-LAEA system and were created with Python 3.7.3 (https://www.python.org) using the Geopandas (v0.6.2, https://geopandas.org/) and Matplotlib (v3.1.2, https://matplotlib.org/) libraries.

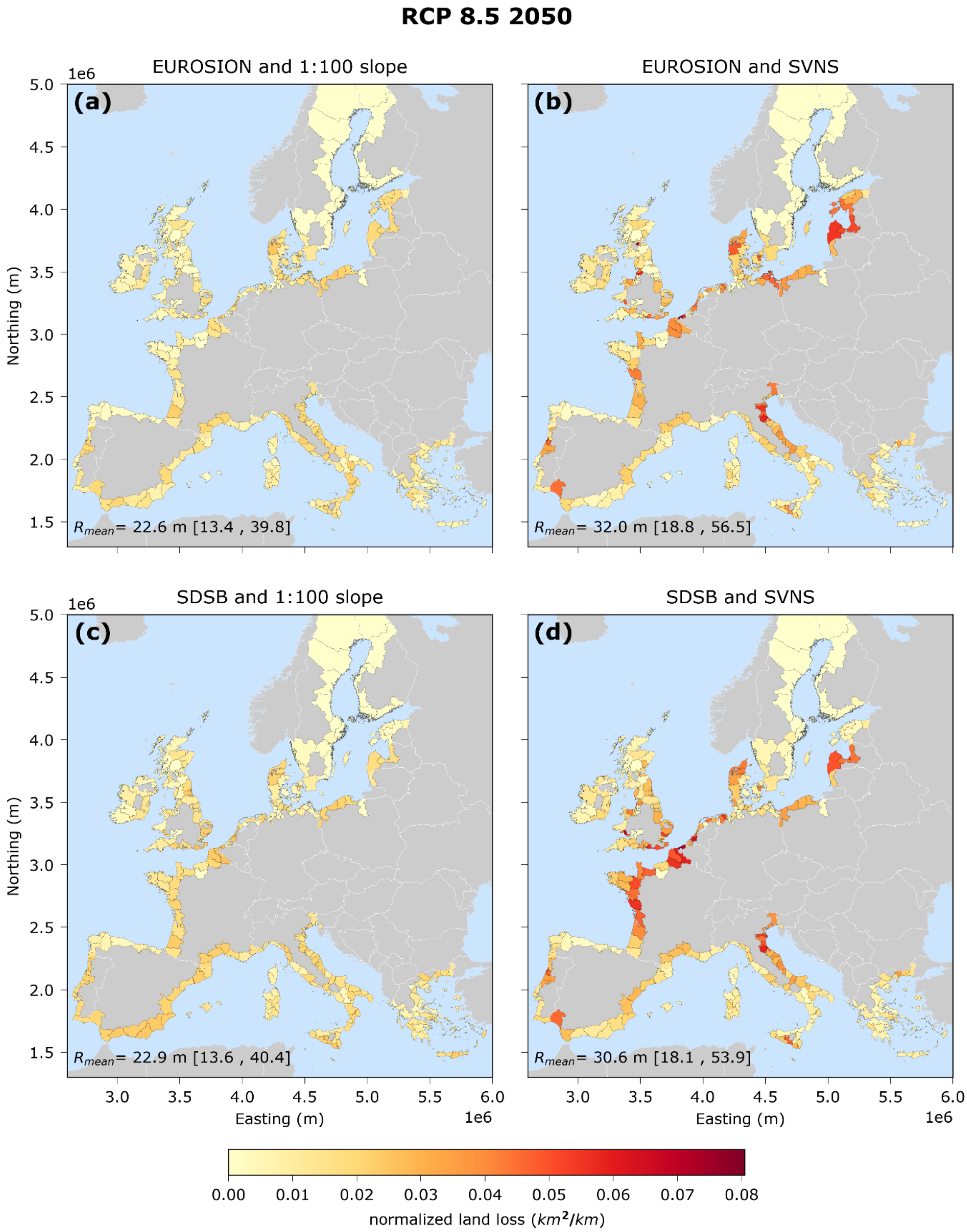

Figure S.3: Normalized coastal land loss (km²/km) projections per NUTS 3 region for the median SLR at 2050 under RCP 8.5 (in the absence of ambient shoreline changes), relative to the baseline year 2010. The coastal land loss has been normalized per the coastline length of each NUTS3 region. Note that each region has a variable area and thus coastline length as defined by Eurostat. Each map represents an assessment with a specific combination of geophysical (sandy beach location and nearshore slope): (a) EUROSION and 1:100 slope, (b) EUROSION and SVNS, (c) SDSB and 1:100 slope and (d) SDSB and SVNS. The values at the bottom left of each map indicate the European average shoreline retreat (m) and are derived for median SLR projections, while in the brackets the mean EU values are given for the of 5th to 95th percentiles of the SLR projections. The maps are projected in the ETRS89-LAEA system and were created with Python 3.7.3 (https://www.python.org) using the Geopandas (v0.6.2, https://geopandas.org/) and Matplotlib (v3.1.2, https://matplotlib.org/) libraries.

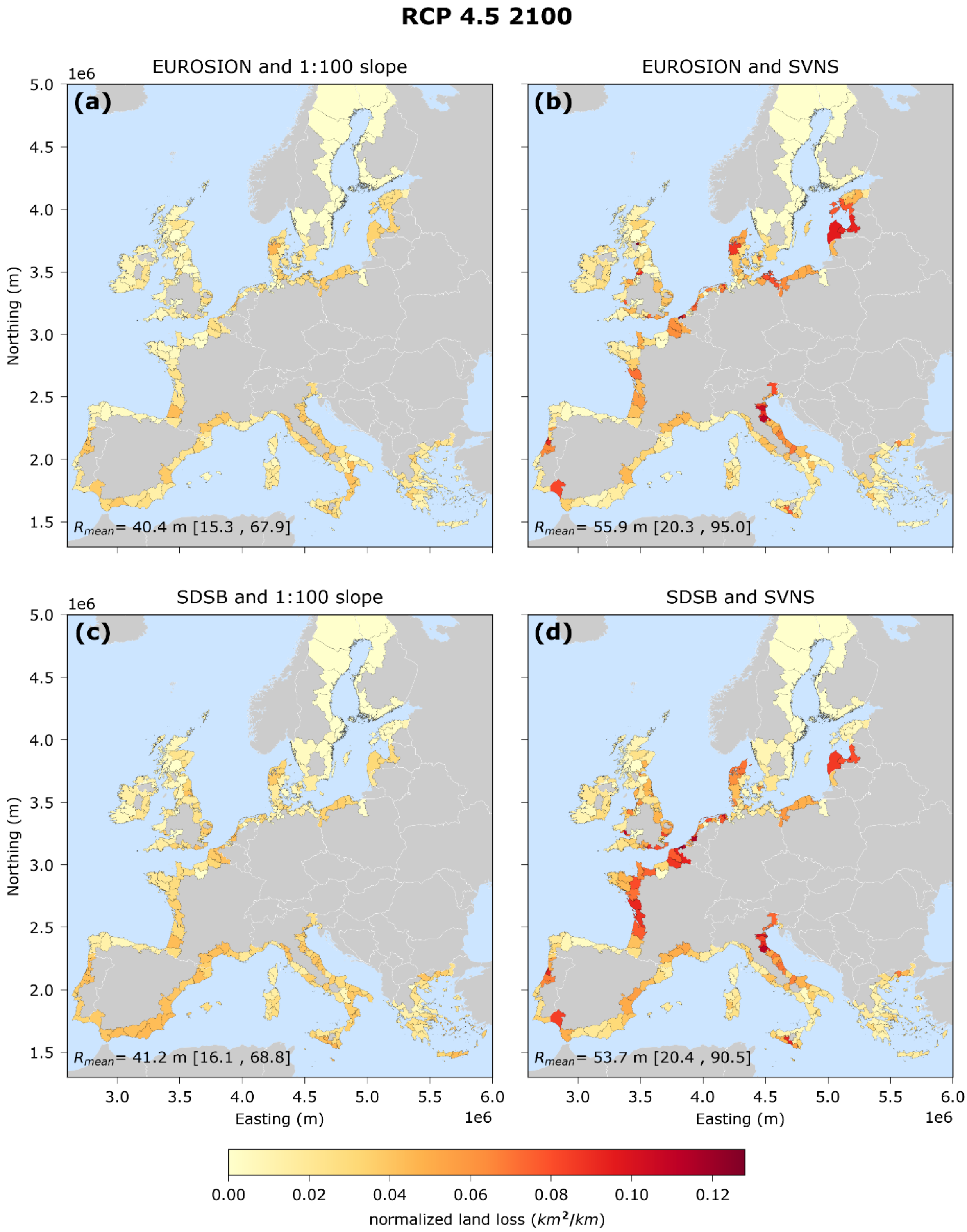

Figure S.4: Normalized coastal land loss (km²/km) projections per NUTS 3 region for the median SLR at 2100 under RCP 4.5 (in the absence of ambient shoreline changes), relative to the baseline year 2010. The coastal land loss has been normalized per the coastline length of each NUTS3 region. Note that each region has a variable area and thus coastline length as defined by Eurostat. Each map represents an assessment with a specific combination of geophysical (sandy beach location and nearshore slope): (a) EUROSION and 1:100 slope, (b) EUROSION and SVNS, (c) SDSB and 1:100 slope and (d) SDSB and SVNS. The values at the bottom left of each map indicate the European average shoreline retreat (m) and are derived for median SLR projections, while in the brackets the mean EU values are given for the of 5th to 95th percentiles of the SLR projections. The maps are projected in the ETRS89-LAEA system and were created with Python 3.7.3 (https://www.python.org) using the Geopandas (v0.6.2, https://geopandas.org/) and Matplotlib (v3.1.2, https://matplotlib.org/) libraries.

2050

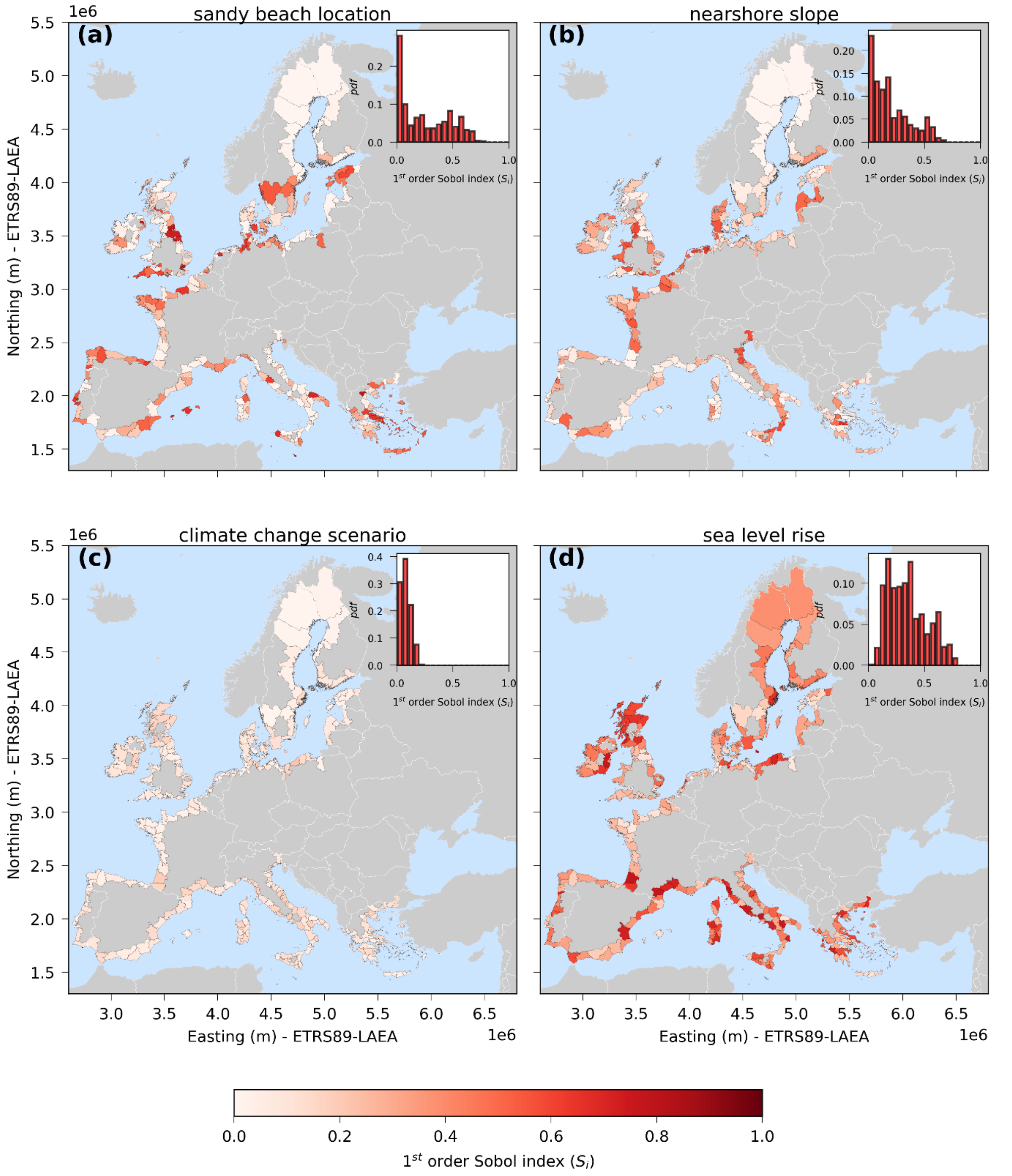

Figure S.5: Map of 1st order Sobol indices value per NUTS 3 region for each unknown parameter at 2050. These are: (a) the sandy beach location, (b) the nearshore slope, (c) the climate change scenario and (d) the sea level rise projections. The histogram of the indices per region, weighted by their relative coastal length, is plotted at the top right corner of each map. The maps are projected in the ETRS89-LAEA system and were created with Python 3.7.3 (https://www.python.org) using the Geopandas (v0.6.2, https://geopandas.org/) and Matplotlib (v3.1.2, https://matplotlib.org/) libraries.

2100

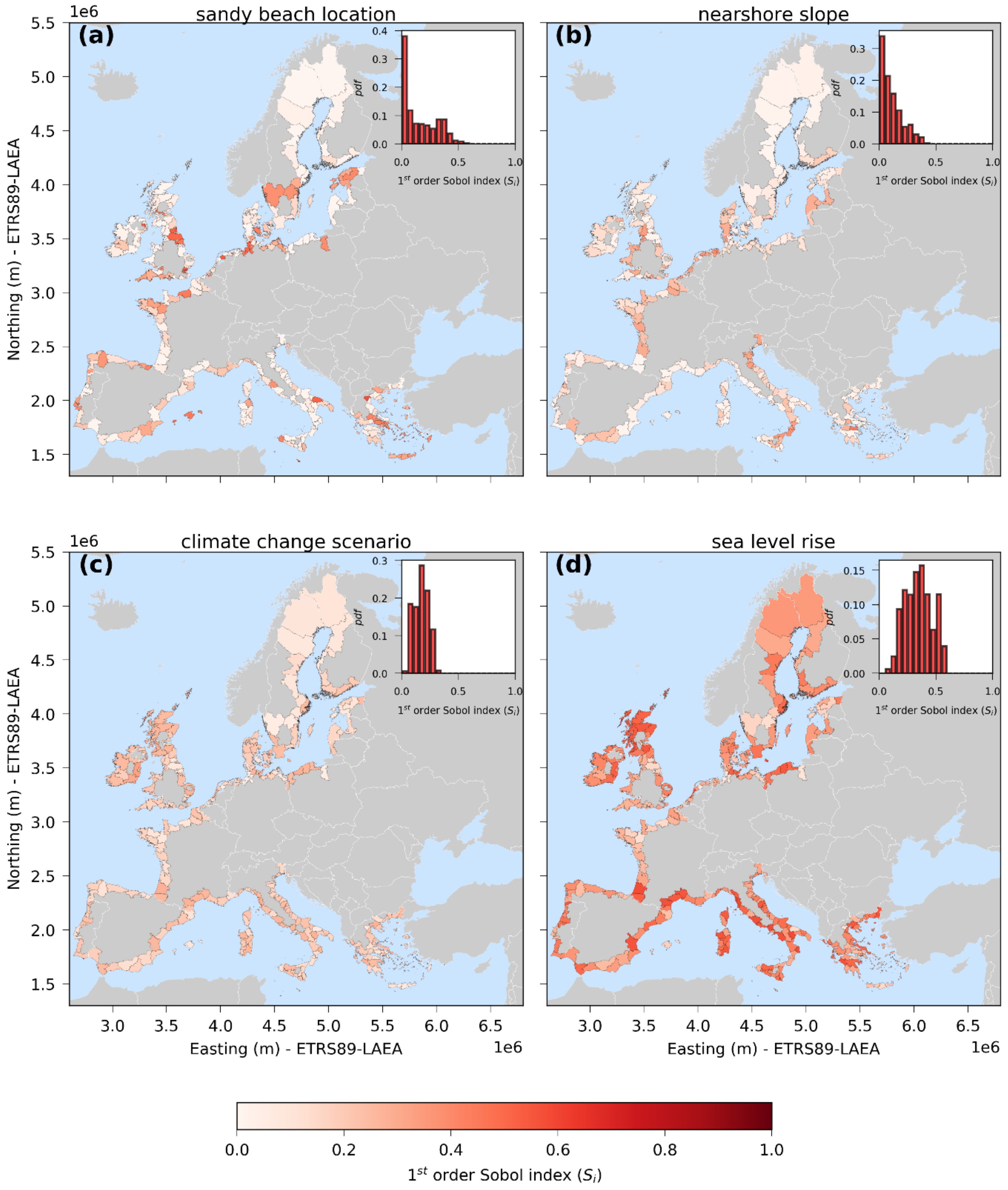

Figure S.6: Map of 1st order Sobol indices value per NUTS 3 region for each unknown parameter at 2100. These are: (a) the sandy beach location, (b) the nearshore slope, (c) the climate change scenario and (d) the sea level rise projections. The histogram of the indices per region, weighted by their relative coastal length, is plotted at the top right corner of each map. The maps are projected in the ETRS89-LAEA system and were created with Python 3.7.3 (https://www.python.org) using the Geopandas (v0.6.2, https://geopandas.org/) and Matplotlib (v3.1.2, https://matplotlib.org/) libraries.

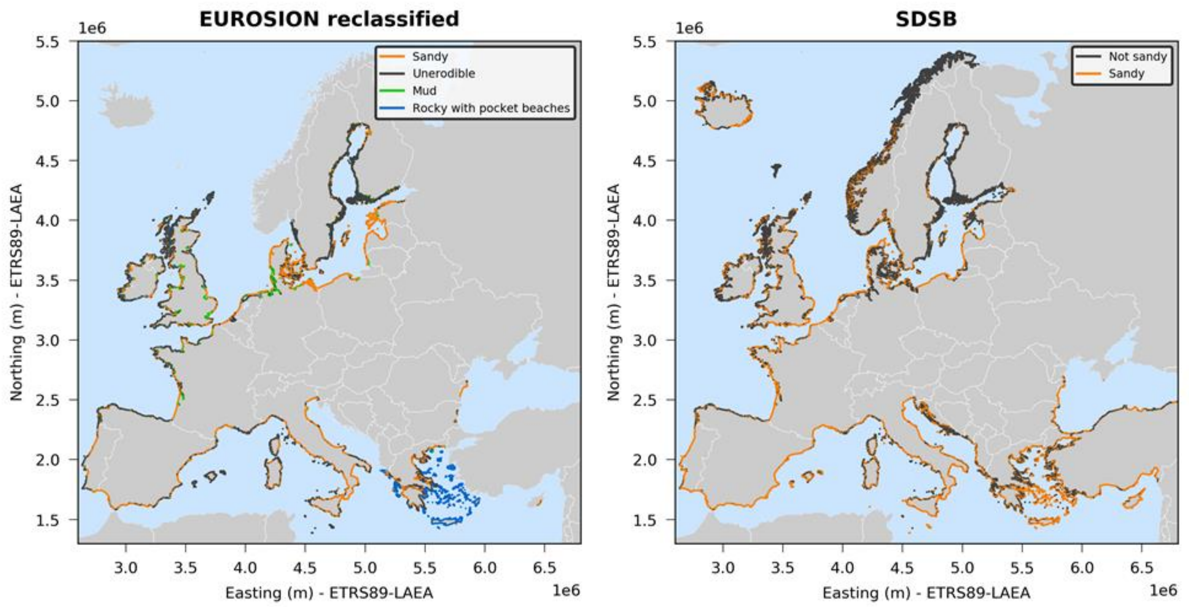

Figure S.7: Map of sandy beach location from: (Left) the reclassification of the EUROSION dataset [1] and (right) the SDSB dataset[2]. The maps are projected in the ETRS89-LAEA system and were created with Python 3.7.3 (https://www.python.org) using the Geopandas (v0.6.2, https://geopandas.org/) and Matplotlib (v3.1.2, https://matplotlib.org/) libraries.

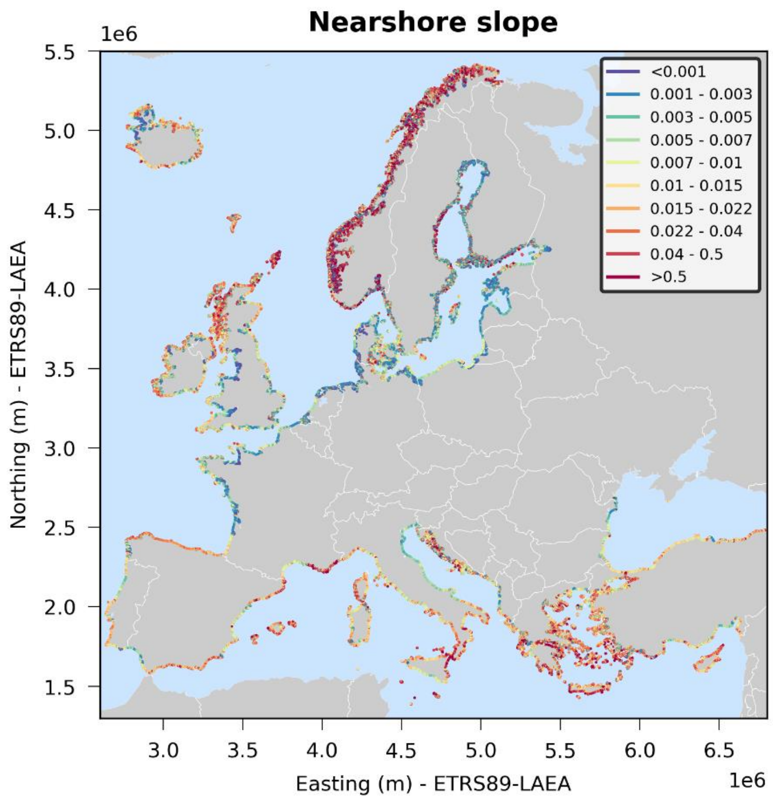

Figure S.8: Map of data-based nearshore slope $tan(\beta)$ around the European coastline [3]. Red colors indicate steep slopes while blue colors, mild slope. The map is projected in the ETRS89-LAEA system and was created with Python 3.7.3 (https://www.python.org) using the Geopandas (v0.6.2, https://geopandas.org/) and Matplotlib (v3.1.2, https://matplotlib.org/) libraries.

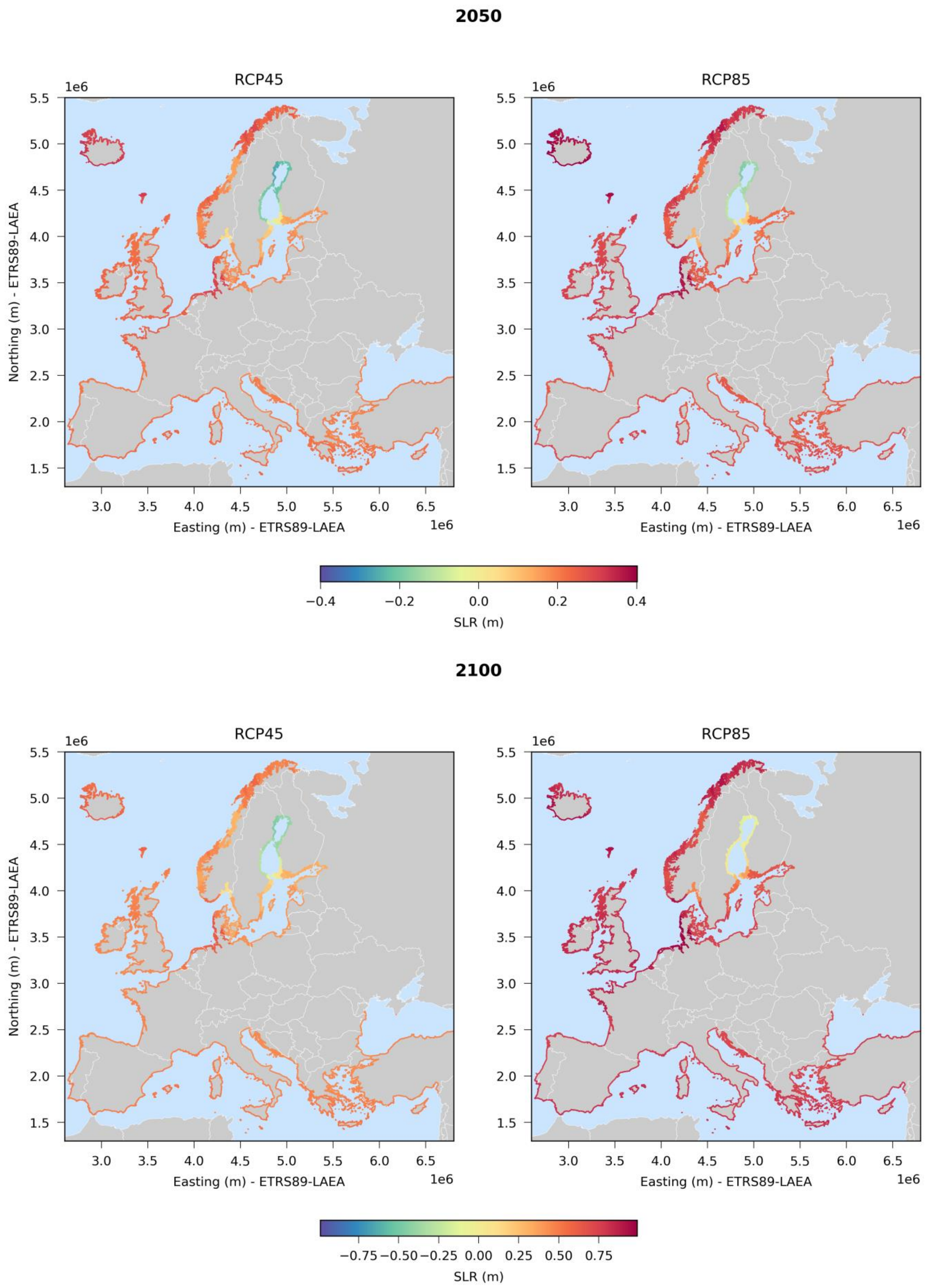

Figure S.9: Maps of the median regional sea level rise (SLR) at each alongshore computational point in Europe after using proximity analysis for the year of 2050 (top panel) and 2100 (bottom panel), relative to 1986–2005, for RCP 4.5 and 8.5 mitigation scenarios[4]. The maps are projected in the ETRS89-LAEA system and were created with Python 3.7.3 (https://www.python.org) using the Geopandas (v0.6.2, https://geopandas.org/) and Matplotlib (v3.1.2, https://matplotlib.org/) libraries.

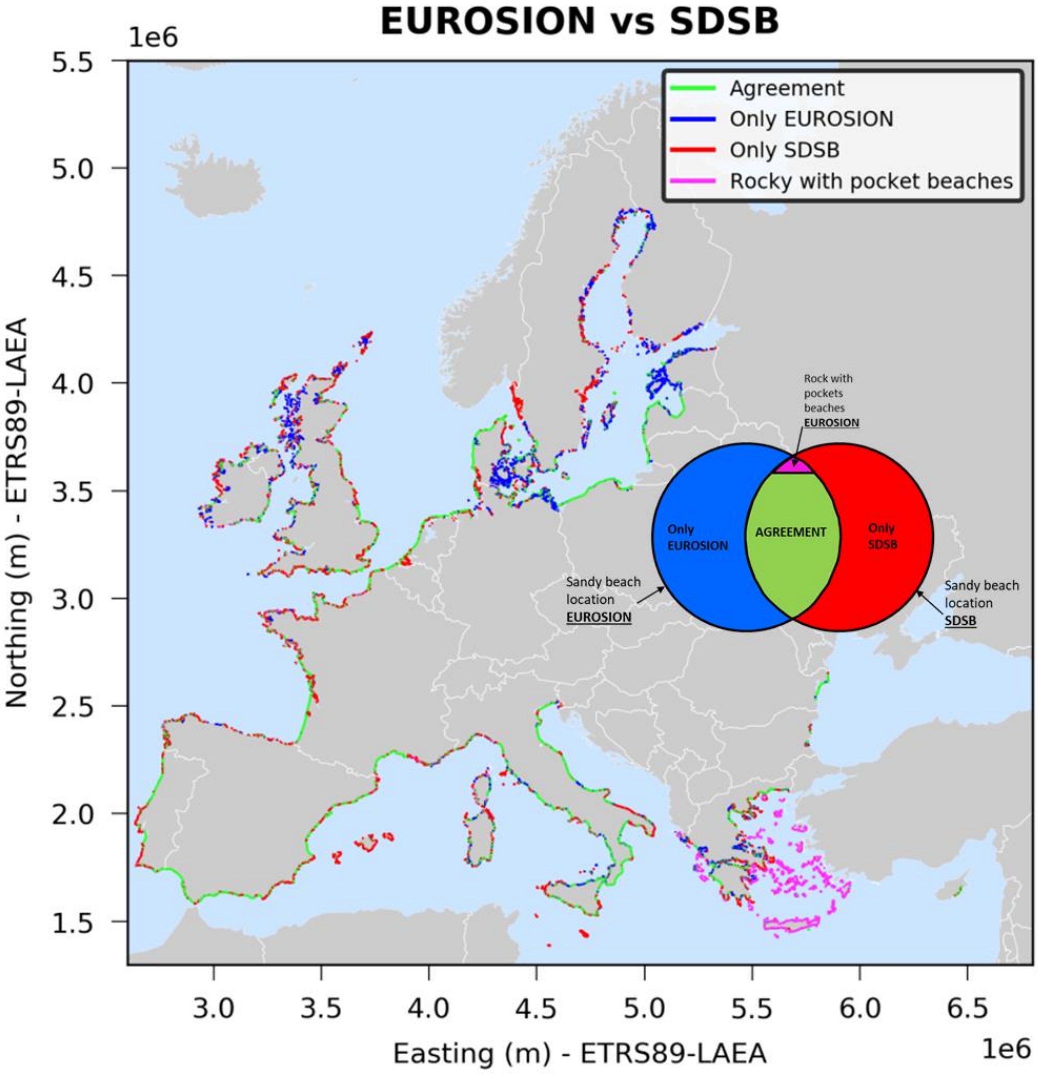

Figure S.10: Vector point agreement between the EUROSION and the SDSB dataset on the coastal classification of sandy beaches. Green points show locations identified as sandy by both datasets, blue points are locations identified as sandy by the EUROSION dataset only, red points are locations identified as sandy by the SDSB dataset only, and purple points are locations identified as sandy by SDSB and as rocky with pocket beaches by EUROSION. The map is projected in the ETRS89-LAEA system and was created with Python 3.7.3 (https://www.python.org) using the Geopandas (v0.6.2, https://geopandas.org/) and Matplotlib (v3.1.2, https://matplotlib.org/) libraries.

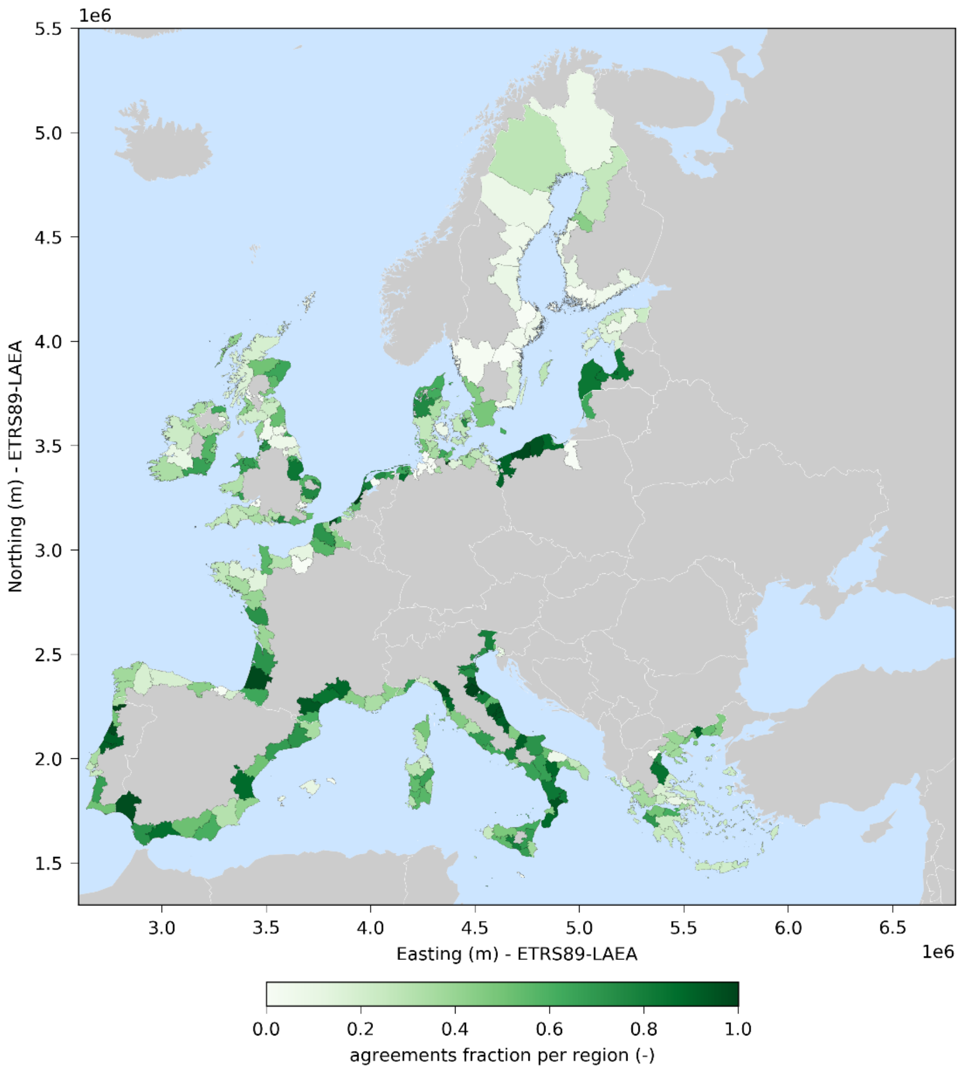

Figure S.11: Map of percentage of sandy beach length agreement between the EUROSION and the SDSB dataset for each NUTS 3 region. The values have been calculated as the part of the coastal locations that both datasets identify as sandy relative to the total sandy grid points of both datasets. The map is projected in the ETRS89-LAEA system and was created with Python 3.7.3 (https://www.python.org) using the Geopandas (v0.6.2, https://geopandas.org/) and Matplotlib (v3.1.2, https://matplotlib.org/) libraries.

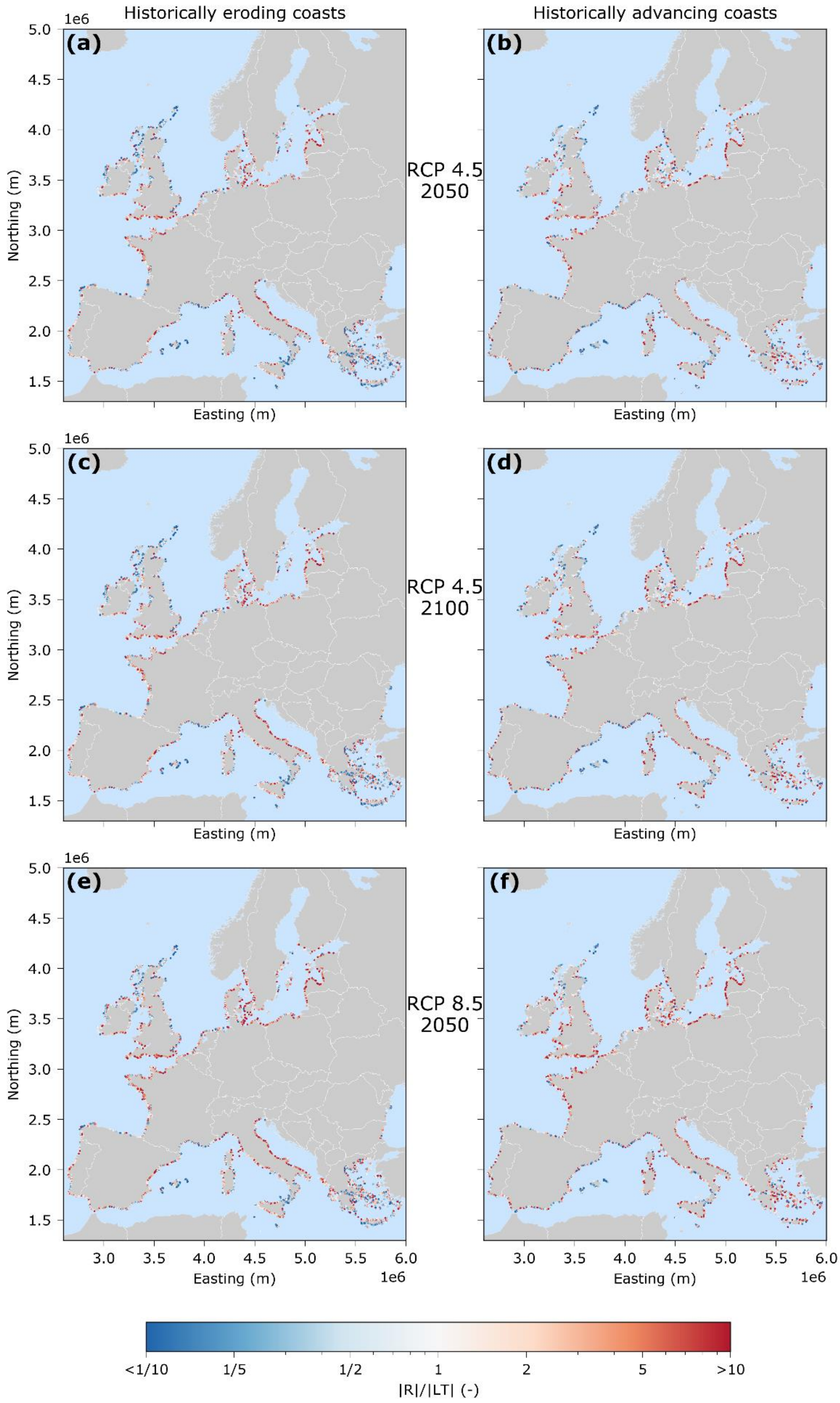

Figure S.12: Ratios of SLR driven shoreline retreat to erosion due to ambient shoreline changes |R|/|AC| for median projected SLR under RCP 4.5 in 2050 (a,b), 2100 (c,d) and under RCP 8.5 in 2020 (e,f) for historically eroding (HE) coasts (a,c,e) and historically advancing (HA) coasts (b,d,f). The maps are projected in the ETRS89-LAEA system and were created with Python 3.7.3 (https://www.python.org) using the Geopandas (v0.6.2, https://geopandas.org/) and Matplotlib (v3.1.2, https://matplotlib.org/) libraries.

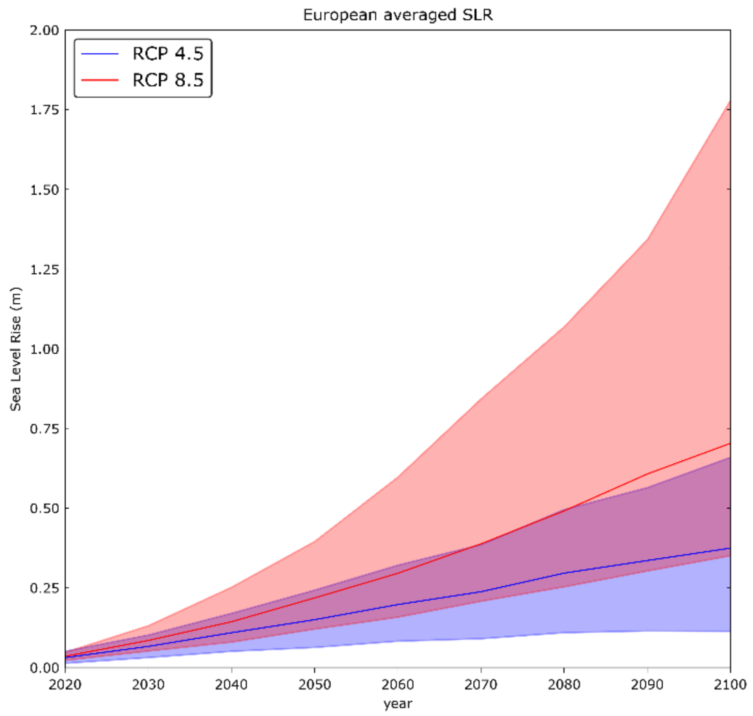

Figure S.13: European averaged SLR for the present century under RCP 4.5 and RCP 8.5 relative to the baseline year 2010. The values are derived by averaging the SLR projection by Vousdoukas et al. 2019 at the offshore points of the European coastline that was studied herein. Shaded areas indicate the 5% and 95% confidence intervals.

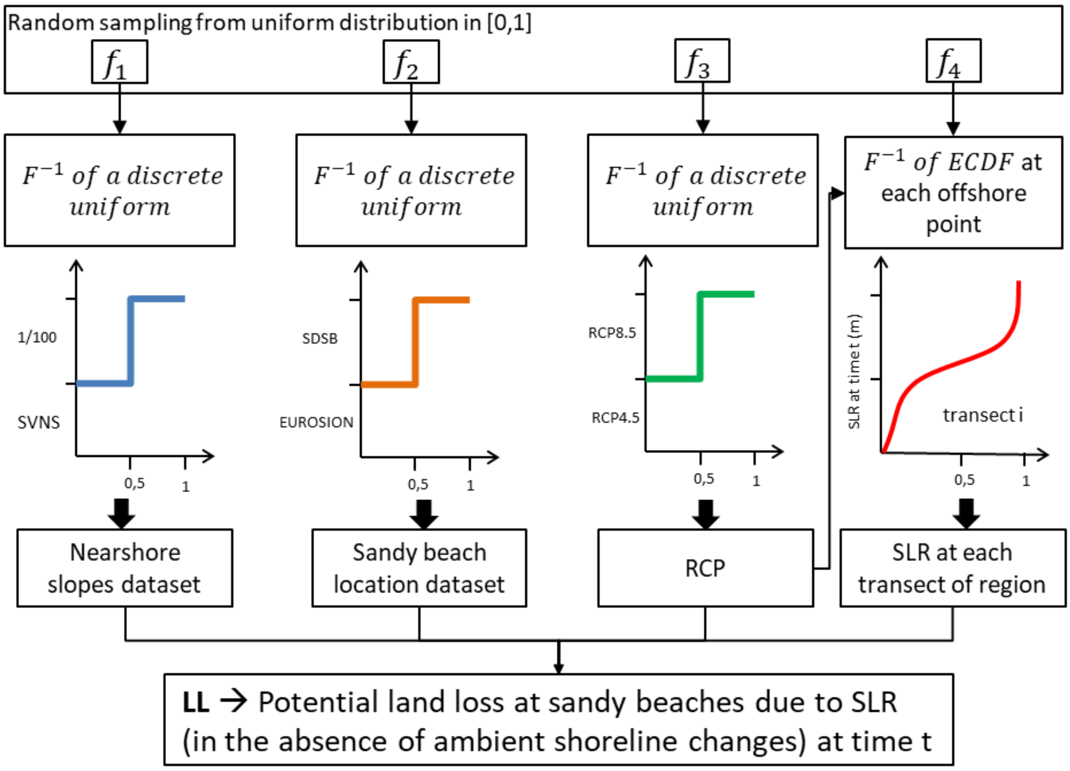

Figure S.14: Uncertainty analysis computational framework for generating random samples of potential coastal land loss for each region and each decade. F^{-1} indicates the inverse cumulative distribution function. This process is repeated 50,000 times, generating 50,000 samples of coastal land loss for each European NUTS3 region and each decade. The input random variables f_1-f_4 are generated following a Sobol sequence of 5,000 quasi-random numbers.

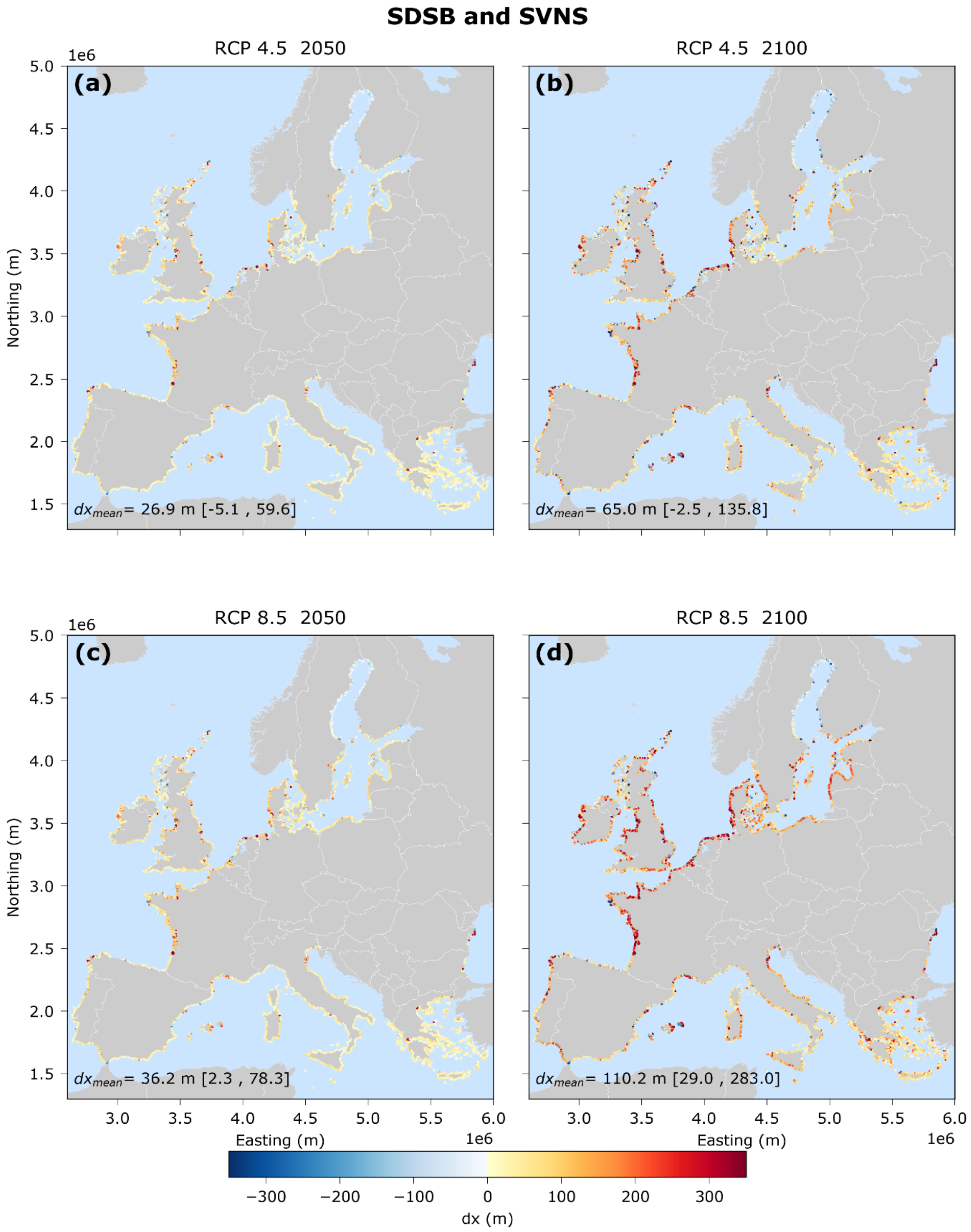

Figure S.15: Median potential shoreline change dx (m) projections at sandy locations under RCP 8.5 (c,d) and RCP 4.5 (a,b) at 2050 (a,c) and 2100 (b,d), relative to the baseline year 2010 including both ambient shoreline changes AC (from Vousdoukas et al. (2020)) and SLR driven shoreline change (R). Positive dx values indicate shoreline retreat, while negative values indicate shoreline advance. Here, dx was calculated in a probabilistic manner using a Monte Carlo approach with 100,000 simulations by sampling from the SLR induced retreat R and the ambient long-term change AC distributions and adding them up. The maps illustrate an assessment using the SDSB sandy beach distribution dataset and the SVNS slopes data. The values at the bottom left of each map indicate the European average potential shoreline change dx (m) and are for median dx projections, while the mean EU values of the of 5th to 95th percentiles of the dx probabilistic projections are given within brackets. The maps are projected in the ETRS89-LAEA system and were created with Python 3.7.3 (https://www.python.org) using the Geopandas (v0.6.2, https://geopandas.org/) and Matplotlib (v3.1.2, https://matplotlib.org/) libraries.

References

1. EUROSION. *Living with coastal erosion in Europe: Sediment and Space for Sustainability: PART I - Major findings and Policy Recommendations of the EUROSION project*. *Service contract B4-3301/2001/329175/MAR/B3 "Coastal erosion – Evaluation of the need for action". Directorate General Environment, European Commission* (2004).
2. Luijendijk, A. *et al.* The State of the World's Beaches. *Sci. Rep.* **8**, 6641 (2018).
3. Athanasiou, P. *et al.* Global distribution of nearshore slopes with implications for coastal retreat. *Earth Syst. Sci. Data* **11**, 1515–1529 (2019).
4. Jackson, L. P. & Jevrejeva, S. A probabilistic approach to 21st century regional sea-level projections using RCP and High-end scenarios. *Glob. Planet. Change* **146**, 179–189 (2016).
5. Vousdoukas, M. I. *et al.* Sandy coastlines under threat of erosion. *Nature Climate Change* **10**, 260–263 (2020).
